# Mechanisms of interventions targeting modifiable factors for dementia risk reduction

**DOI:** 10.1186/s13024-025-00845-w

**Published:** 2025-06-23

**Authors:** Anna Matton, Ruth Stephen, Makrina Daniilidou, Mariagnese Barbera, Vilma Alanko, Marcel Ballin, Jamie Ford, Katri Hemiö, Jenni Lehtisalo, Sabsil López Rocha, Francesca Mangialasche, Tiia Ngandu, Anna Rosenberg, Gazi Saadmaan, Chinedu Udeh-Momoh, Kerttu Uusimäki, Alina Solomon, Miia Kivipelto

**Affiliations:** 1 Division of Clinical Geriatrics, Department of Neurobiology, Care Sciences and Society, Karolinska Institutet, 171 76 Stockholm, Sweden; 2 Division of Neurogeriatrics, Department of Neurobiology, Care Sciences and Society, Karolinska Institutet, 171 77 Stockholm, Sweden; 3 Ageing Epidemiology (AGE) Research Unit, SchoolofPublicHealth, Imperial College London, London, W6 8RP UK; 4 FINGERs Brain Health Institute, 171 76 Stockholm, Sweden; 5 Institute of Clinical Medicine/Neurology, University of Eastern Finland, 702 10 Kuopio, Finland; 6 Department of Public Health and Caring Sciences, Clinical Geriatrics, Uppsala University, 751 22 Uppsala, Sweden; 7 Department of Public Health, The Lifestyles and Living Environments Unit, Finnish Institute for Health and Welfare, 003 00 Helsinki, Finland; 8 Institute of Public Health and Clinical Nutrition, University of Eastern Finland, 702 10 Kuopio, Finland; 9 Theme Inflammation and Aging, Karolinska University Hospital, 171 76 Stockholm, Sweden; 10 School of Public Health Sciences, Wake Forest University School of Medicine, Winston-Salem, NC United States of America; 11 Brain and Mind Institute, Aga Khan University, Nairobi, Kenya; 12 Sheffield Institute for Translational Neuroscience (SITraN), University of Sheffield, Sheffield, UK

## Abstract

The global prevalence of dementia is increasing. With no widely available and accessible treatments to halt or reverse the progression of dementia, exploring preventative strategies is critical. Lifestyle-based interventions show promise in preventing or delaying dementia onset. However, understanding the complex and multifactorial mechanisms underlying dementia, and how interventions target these pathways, is essential for developing personalized and effective strategies. In this review, we examined the current evidence of the mediating pathways in dementia risk reduction. We focused on mechanisms investigated in single-domain interventions on physical exercise, cognitive training, diet, metabolic/cardiovascular or psycho-social risk factors in line with those combined in the landmark FINGER trial. Additionally, we synthesized existing literature on mechanisms of action in multimodal interventions combining multiple lifestyle changes. Most evidence was identified in relation to neuroimaging biomarkers with positive effects for all intervention components. The evidence among fluid biomarkers of Alzheimer’s disease and related disorders (ADRD) (amyloid-beta peptide (Aβ), tau and neurofilament light chain (Nfl)) vascular markers, inflammatory markers, and neurotrophins were less conclusive, though physical exercise consistently appeared to impact several of these pathways. The findings of this review underscore the potential of lifestyle-based interventions in modulating several different types of pathophysiological pathways associated with dementia. As the number of dementia cases reach epidemic proportions, a multifaceted approach is needed. We propose that the next critical step in dementia prevention/risk reduction is to refine existing intervention tools and develop an adaptive platform that integrates different lifestyle interventions tailored to individual risk profiles and needs. Understanding the underlying mechanisms and biomarkers related to modifiable risk factors will be instrumental to optimising these interventions.

**Supplementary Information:**

The online version contains supplementary material available at 10.1186/s13024-025-00845-w.

## Background

Despite recent evidence of a decreasing trend in incidence of dementia in high-income countries [1], more than 150 million people globally are estimated to be affected by 2050 [2] pre-dominantly in low-middle-income countries. Alzheimer’s disease (AD) is the most common cause of dementia, accounting for 60–70% of cases worldwide [3, 4]. The recently-reported phase-III trial findings for amyloid β peptide (Aβ)-targeted monoclonal antibodies [5–7] have raised hopes for the first disease-modifying therapies (DMTs) in AD being broadly available in the near future, especially for those in the early stages of the disease. However, an intense debate within the scientific community is currently ongoing [8] around their inconclusive risk–benefit profiles; unknown long-term effects; and high costs [9]. This is also translating into inconsistent decision-making and market approval status by various drug regulatory authorities [10], making the availability of these medication unequal world-wide. Furthermore, even in countries where such medications are available and provided by the national healthcare system, only a proportion of AD patients may be eligible for these novel treatments [11]. The benefits could be further limited due to the complex nature of AD [12] and common prevalence of concomitant mixed pathology [13].

The diverse pathogenesis and disease progression may vary among dementia patients depending on their genetic background, lifestyle, and environmental conditions. Due to the strong links between modifiable risk factors and dementia, growing efforts are being directed towards prevention and risk reduction. Indeed, the World Health Organization has emphasized prevention as a key element to address the’dementia epidemic’ [14]. It has been suggested that modifiable risk factors account for approximately 45% of worldwide dementias, providing significant opportunities to reduce the number of affected individuals [15]. The risk factors are believed to act in varying extent throughout life although their role may extend and cumulate during the whole lifespan.

In general, brain pathology progresses gradually over time, beginning with an asymptomatic period, followed by a prodromal phase of mild cognitive impairment (MCI), and eventually, dementia [16]. The gradual development provides a window for early preventive interventions, before the clinical onset of dementia. The multifactorial nature of the disease provides opportunities for prevention with multimodal risk reduction (with or without DMT) targeting dementia at-risk populations [17]. The first three large multimodal trials reported inconsistent findings [18–20]. Among them, the Finnish Geriatric Intervention Study to Prevent Cognitive Impairment and Disability (FINGER) was the only one to show positive effects on cognition and other relevant outcomes. The FINGER model, including dietary guidance, physical exercise, cognitive training, vascular/metabolic risk monitoring and social activities is currently being tested in the World-Wide FINGERS project (WW-FINGERS) [21, 22].

**Fig. 1:**
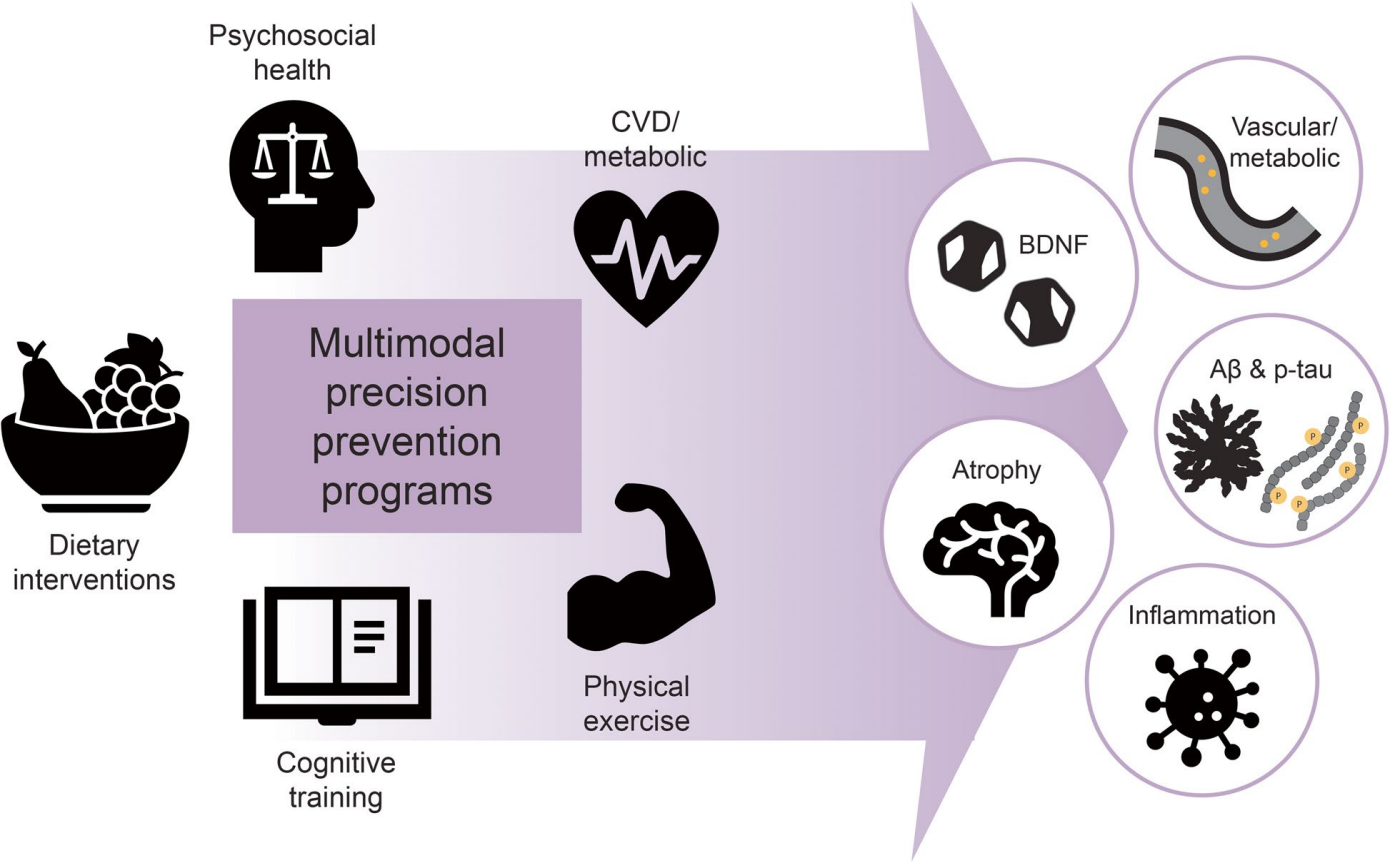
Schematic overview of risk reduction strategies, and the main dementia-related mechanisms and mediators that have been investigated in the context of lifestyle intervention RCTs and included in this review. Abbreviations: BDNF: brain-derived neurotrophic factor; Aβ: amyloid beta peptide; p-tau: phosphorylated tau protein

In this review we provide an overview of the current landscape of single- and multimodal human intervention studies emphasizing their relationship to neuroimaging and fluid biomarkers. We explore studies with the same intervention domains as those targeted in the FINGER model (Fig. 1 shows the included intervention modalities and studied mechanisms). Furthermore, we summarize studies on the combined effects of multiple domains on biomarkers associated with dementia and aging (i.e., β-amyloid, tau, neuroinflammation, brain atrophy, vascular/metabolic function, neurotrophins). Finally, we address current knowledge gaps and outline recommended future directions for prevention and/or risk reduction of dementia.

## Methods

This is a narrative review of available evidence from studies investigating the underlying biomarkers and mechanisms of lifestyle-based interventions for dementia risk reduction. Eligible studies were those performed in the entire continuum before dementia onset, including healthy older people, at-risk populations, MCI and prodromal AD which participated in trials aiming for dementia risk reduction. The review is structured according to the following sections each representing a lifestyle intervention approach: Physical exercise, cognitive training, dietary interventions, interventions targeting cardiovascular and metabolic factors, psychosocial health and finally multimodal interventions.

**Fig. 2:**
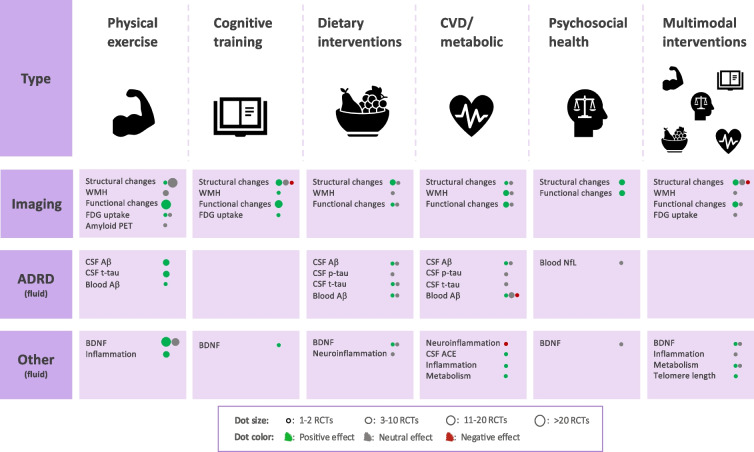
Summary of results. Type of biomarkers are listed in the left panel (Imaging, ADRD fluid biomarkers and Other fluid biomarkers). Columns represents the intervention domains included in this review (Physical exercise, Cognitive training, Dietary intervention, CVD/metabolic risk reduction, Sleep/Mindfulness with Psycho-social health and Multimodal intervention). The number of studies are indicated by dot size and colour represents positive (green), neutral (grey) or negative (red) results from the trials

A systematic-like approach was applied to identify the relevant literature for this review, with pre-defined keywords for each dementia preventive component used to search the literature in PubMed. Outcome keywords were kept consistent throughout the searches and included both fluid- and neuroimaging biomarkers (a complete list of search terms can be found in Supplementary Table 1). Searches were filtered to be English language and from year 2000. Observational studies were included only when randomized controlled trials (RCTs) were absent. Biomarkers commonly studied in dementia/ADRD were prioritized, still less studied biomarkers were included too, especially for the domains with fewer available biomarker studies in general. The articles discussed are listed by intervention type in Supplementary Tables 2, 3, 4, 5, 6, 7, 8, 9 and 10) where details on intervention design, included populations, biomarker outcomes and main findings for each trial can be found. The key findings summarized from all the included RCTs are illustrated in Fig. 2. Overviews of included RCTs and number of participants in each domain are shown in Supplementary Fig. 1.

## Dementia risk reduction by physical exercise

Exercise is recognized as one of the most effective risk reduction strategies against cognitive decline and dementia [15]. Numerous interventional studies have explored its impact on both neuroimaging and fluid biomarkers (included studies listed in Supplementary Table 2). A summary of the key findings is illustrated in Fig. 2 and is further elaborated in the sections below.

### Physical exercise: associations with neuroimaging biomarkers

Brain structure and function have been studied in relation to exercise using a variety of neuroimaging modalities. Structural magnetic resonance imaging (MRI) studies reported mixed results [23, 24]. Few trials assessing the impact of short-term (3–12 months) aerobic exercise on adults without substantial neuropathology observed increases in regional brain volumes [24, 25] and in MCI protective effects from exercise were seen in hippocampal volume [26]. However, other studies in MCI patients reported no significant effects on the brain structure itself via physical exercise [27–29]. The discrepancy may be partly due to the type of exercise and duration, or the baseline level of pathology which may impact one’s ability to exercise, or the benefits of exercise.

The brain white matter is sensitive to neuropathological changes and ageing and is considered an early marker of AD [30]. In active, healthy older adults, high or moderate-intensity exercise did not show a significant effect on the white matter integrity [31–34]. This may be attributed to the already active lifestyle of the trial participants, and therefore, not leaving much room for improvement. In individuals with memory complaints and MCI, long-term (24 months) moderate-intensity exercise did not affect the progression of white matter hyperintensities (WMH; disruption in myelin commonly associated with vascular lesions) [33]. This may be attributed to the extent of pre-existing pathology or perhaps the need for a more intensive and sustained lifestyle intervention including a comprehensive pharmacological approach. However, smaller effects on white matter integrity in prefrontal and temporal lobes prone to pathological insults have been observed in sub-groups of individuals with increased cardiorespiratory fitness after an exercise intervention [31].

Functional MRI measures in healthy older individuals, show significant regional activation among exercisers within multiple sub-regions of the temporal and frontal lobes, as well as other subcortical regions [35, 36]. Modest improvements have also been shown in other in vivo factors such as carotid arterial stiffness, cerebral blood flow (CBF) and oxygenation [37–40]. Increased brain glucose uptake using 18-fluorodeoxyglucose positron emission tomography (18-FDG-PET) was seen during rest within the caudate nucleus [41], and parietal and temporal lobes among healthy younger and older adults engaging in high-intensity exercise [42]. Another smaller trial observed no significant effect on increasing brain glucose uptake [43] however this may be due to the lower intervention intensity.

### Physical exercise: associations with ADRD fluid biomarkers

Exercise has been shown to impact ADRD fluid biomarkers; however, the disease stage may influence the effects. Patients in the dementia continuum performing Baduanjin (a traditional Chinese exercise that combines breathing, body movement, meditation, and awareness) showed improved cognition and a significant improvement of both tau and Aβ_1–42_ levels in the cerebrospinal fluid (CSF) compared to standard care [44]. On the other hand, for older adults without cognitive impairment, aerobic exercise intervention of moderate intensity and length did not influence global amyloid burden [45], indicating that Aβ-pathology may not be directly influenced by exercise per se*,* but rather through reducing other (albeit related) disease mechanisms such as inflammation and oxidative stress. Whether tau pathology is directly affected by exercise or as downstream factor, has not been explored to our knowledge in RCTs (especially those studying people without dementia). Animal studies do provide some evidence that exercise is implicated in lowering tau levels. However, observational evidence from human studies is sparse and less consistent [46] and highlights the need for robust exercise-focused RCTs having tau as outcome.

Among the more recently studied biomarkers, e.g., neurofilament light chain (NfL, a marker of axonal damage) there are studies suggesting that exercise slows cognitive decline among older adults with increased NfL together with higher total tau concentrations in the blood [47, 48]. The precursor protein of the Aβ-peptide, amyloid precursor protein (APP), with a physiological function believed to be related to neuroprotection and synaptic plasticity, was observed to be increased from a dance training intervention [49] yet the downstream effect on Aβ levels was not determined.

### Physical exercise: associations with BDNF and other fluid biomarkers

Neuroinflammation has been shown to play a significant role in the neurodegenerative cascades [50] and exercise is thought to influence these by regulating microglia, inhibiting pro-inflammatory cytokines, and supporting neuroprotective factors [51]. A meta-analysis of 13 RCTs on participants with MCI showed that exercise had positive effects by decreasing the pro-inflammatory molecules TNF-α and C-reactive protein and promoting neuroprotective factors such as brain derived neurotrophic factor (BDNF) and insulin-like growth factor (IGF-1) [52]. On the other hand, depending on the intensity and type of exercise, skeletal muscles may also react in opposite direction by inducing the release of pro-inflammatory cytokines (e.g., interleukin (IL)−6, IL-10, tumour necrosis factor α (TNF)-α) [53, 54] and by stimulating glial activation and inflammation [55, 56]. Thus, the relationship between exercise and inflammation is multi-faceted.

With respect to neuroprotective mechanisms, the most studied outcome is BDNF. BDNF is a key molecule involved in neuroplastic changes related to learning and memory. BDNF plays an important role in differentiation and maturation of neurons and maintains high expression levels regulating both excitatory and inhibitory synaptic transmission and activity-dependent plasticity [57]. Interestingly, pharmacological compounds such as Memantine and Donepezil used for the management of AD symptoms also markedly increase BDNF levels in a dose-dependent manner [58, 59]. Physical exercise has direct effects on the production and secretion of myokines such as BDNF [60–62] which act as mediators in the muscle-brain crosstalk [56]. Specifically, high-intensity exercise has been reported to have immediate impact on serum BDNF levels in exercisers compared to older adults who had little or no physical exercise [63–67]. When considering exercise frequency, intensity, and type, both acute and long-term exercise had significant positive effects on BDNF levels [68–70, 64]. Similarly to the connection between aerobic exercise and altered BDNF levels, strength training has also been shown to increase blood BDNF concentrations [71]. However, there are also studies which do not report a significant effect on BDNF concentrations following exercise intervention in older adults nor patients with MCI [72–76] highlighting the need to identify the right target groups for each intervention type.

The serotonergic pathway may also be implicated in neurocognitive disorders; including dementia. For example, the relationship between physical exercise and serum serotonin levels is moderated by the intervention length and intensity, whereby trials of shorter duration and intensive exercise resulted in an increase in participants’ serotonin levels. Conversely, some longer-term exercise interventions have reported a decrease in serotonin [77–79]. Decreased serotonin levels mimic the effects of selective serotonin reuptake inhibitors whereby low levels in plasma are believed to reflect higher brain serotonin levels [39]. However, the exact biological mechanisms underlying these effects are still not fully understood [78].

## Dementia risk reduction by cognitive training

Strategies to engage the brain and enhance mental function through the specific implementation of cognitive training have been investigated in the context of dementia prevention and risk reduction. Cognitive-based interventions may involve a direct application of structured tasks designed to target or enhance specific cognitive functions, such as memory [80, 81] and pragmatics [82]. However, these interventions may well have broader effects, improving global cognition or enhancing multiple cognitive domains [83–85]. Studies on biomarkers and cognitive training have produced mixed results (RCTs included in this review are listed in Supplementary Table 3). The main findings are summarized in Fig. 2 and described in the biomarker sections below.

### Cognitive training: associations with neuroimaging biomarkers

Some data suggest that improvements in cognitive abilities, facilitated by cognitive training, can elicit beneficial effects on brain structural- [86, 87] and functional plasticity [85, 88, 89] or both [90]. These interventions may also enhance neural plasticity, as evidenced by changes in electroencephalogram readings [91]. Reported improvements include alterations in biomarker profiles such as brain vasculature [90, 92], metabolic indices [84], neurotrophic factors and markers of biological aging [93]; even in persons at risk for AD [94, 95]. Macroscopic resting-state networks based on measures of functional connectivity may serve as a correlate for estimating effect of cognitive training in vivo [96–98]. For example, in cognitively unimpaired older adults, increased functional connectivity between sub-regions of the frontoparietal control network was linked to improved attention and processing speed following a cognitive training intervention [99]. Additionally, connectivity of the cingulo-opercular network has been associated with the same cognitive domains in a similar participant demographic [100]. Notably, brain regions that connect with numerous diverse networks [101] also showed associations with participants improvement in cognitive domains, such as episodic working memory and executive function. Likewise, cognitive training interventions have been linked to global increases and selective plasticity in the associated brain structures linked to cognitive performance, specifically orbito-frontal cortical, post-parietal and medial temporal lobe areas [82]. Furthermore, cognitive training has shown benefits for structural outcome measures, e.g., on neuroimaging biomarkers such as gray matter [86] and hippocampal volumes [26] white matter microstructure [102–105] and cortical thickness [106].

However, other studies have reported nil/negligible impact of cognitive training on brain structural and functional outcomes (e.g., [107] as well as brain health outcomes [94, 106, 108]. In some of these studies, participants with already established neurodegenerative disease were included, suggesting that temporal manifestations in the efficacy of cognitive training with prevention or symptom-progression is a key feature for this mode of intervention. 

### Cognitive training: associations with BDNF

Evidence on the effect of cognitive training on fluid-based biomarkers of neuronal plasticity is quite limited. Nevertheless, positive findings have been reported for cognitive training on blood-based markers of i.e., serum BDNF. Significant improvements in cognitive outcomes seen among amnestic MCI patients following both group and home-based cognitive training interventions were associated to BDNF levels [109].

It is evident that cognitive training and stimulation show promise as significant contributors to dementia prevention through cumulative effects driving brain plasticity that could potentially delay the onset of cognitive decline, however the timing and right target groups should be more investigated.

## Dietary intervention as a dementia risk reduction approach

The role of a healthy diet in the prevention of dementia is well known, but a more detailed understanding of the most beneficial dietary factors depending on age and disease stage is warranted. It also remains unclear whether the positive effects of diet are directly involved in the pathological processes of dementia or through the beneficial changes in other risk factors (e.g., reducing hypertension, hyperlipidemia, obesity, etc.). Both scenarios are plausible, as dietary patterns, foods, and nutrients are known to have direct neuroprotective functions, e.g., as antioxidative or anti-inflammatory molecules or acting through gut microbiota while being also associated with cardiovascular disease (CVD) risk factors for dementia [110].

Food-based interventions investigating pathology-related biomarkers are scarce. In most studies, diet was combined with interventions involving other modifiable risk factors, especially physical exercise [111] and are often conducted primarily to target weight loss [112]. Below we outline the main biomarker findings from the included dietary interventions (Supplementary Table 4. Summary in Fig. 2).

### Dietary interventions: associations with neuroimaging biomarkers

Structural MRI was investigated as a secondary outcome in the intervention studies with largest sample size and longest duration [113, 114]. The MIND-diet (Mediterranean-DASH Intervention for Neurodegenerative Delay) is a hybrid between the Mediterranean diet (MeDi) and the Dietary Approaches to Stop Hypertension (DASH) antihypertensive diet. When the MIND-diet was combined with weight loss and compared with weight loss alone in cognitively healthy, overweight older adults, both groups improved their cognition and no differences in brain volume or WMH were found between groups [113]. Both groups lost significant amounts of weight. Further, an increase in WMH and decreased hippocampal and cortical volumes were seen for both groups, suggesting no benefit of the overall weight loss either. Still, a smaller MIND-focused weight loss intervention among obese women suggested a benefit of the MIND-diet intervention on the inferior frontal gyrus compared with weight loss alone [115].

In the multinational LipiDiDiet study, which assessed the effects of a complex multi-nutrient product (medical food) in participants with prodromal AD, there was significantly less decrease in hippocampal volume and increase in ventricular volumes in the intervention group at the end of the trial [114]. Similar results were found when investigating cognitively healthy people of all ages with cardiovascular disease (CVD)-risk factors: Dietary counselling targeting calorie-restricted MeDi with complementary walnuts resulted in a lower decline in hippocampal size compared with regular healthy diet counselling [116]. Effects were more pronounced among those above 50 years of age and in a MeDi group having more emphasis on plant-based food and additional complementary foods rich in polyphenols. Cerebral perfusion was furthermore improved in people with normal cognition following MeDi compared with Western-type diet [118]. When a palaeolithic diet was compared to standard Nordic Nutrition Recommendations, however, there were significant changes in brain activity measures (fMRI) in both groups but no group differences [117].

### Dietary interventions: associations with ADRD fluid biomarkers

Macronutrient composition-mediated modifications on CSF biomarkers have only been reported in smaller and shorter studies [118–120]. Although the results are not coherent between studies, it is often suggested that the effects may differ depending on disease stage. In the studies including participants with normal cognition, a diet low in both fat and glycemic index (GI) worsened the CSF Aβ42 levels compared with a diet high in fat and GI. Among those with MCI, however, a low fat/low GI diet had beneficial effects on CSF Aβ42 levels [119]. It was hypothesised that these reverse associations would indicate positive effects of diet among both cognitively normal and MCI groups, since there can be stage-dependent differences in the trajectories of CSF Aβ42 in healthy middle and older adult age, pre-symptomatic, and symptomatic MCI and AD. A similar study reported a significant interaction between cognitive status and diet [118]. However, in this case, the ratio CSF Aβ42/40 was improved in the normal cognition group following the MeDi, and worsened after following the Western diet, while the MCI group showed a reverse pattern [118]. Additionally, no difference in CSF Aβ42 was reported, but only Aβ40 decreased after the MeDi diet among those with normal cognition. Lipid-depleted amyloid in CSF was found to be increased by a diet high in fat and GI in both healthy and cognitively impaired individuals [120]. Furthermore, participants at the MCI stage following MeDi showed increased total tau (t-tau) in comparison to decreased t-tau after a Western diet, indicating a benefit of the Western type. This study found no effect of diet on t-tau in the group with normal cognition [118]. In addition to diet-level interventions reviewed here in more detail, smaller studies suggested benefit of vitamin D supplements among vitamin D insufficient adults on plasma Aβ40 [121], but no effect of omega-3 supplementation on CSF Aβ42 in patients with mild to moderate AD [122] or in healthy middle-aged adults [123]. Also, evidence from observational studies supports the association between Mediterranean-styled dietary pattern and other healthy dietary patterns and a beneficial effect on AD biomarkers and subsequent pathology [124, 125].

### Dietary interventions: associations with BDNF and other fluid biomarkers

Low fat/low GI diet has had divergent results depending on disease stage—while CSF insulin levels were increased in people with MCI, levels were lowered in healthy older adults [119]. Plasma BDNF levels have been evaluated in two studies, but the one with a larger sample size and longer duration did not have a baseline assessment of BNDF, however, counselling for MeDi with supplementary Nuts (a subsample of the PREDIMED study [126]) was associated with a decreased risk of low BNDF levels. A smaller study found a slight, non-significant difference in BNDF after a MIND-focused weight-loss intervention [115].

## Dementia risk reduction targeting cardiovascular and metabolic factors

Arteriosclerosis, cerebrovascular blood flow, and neuroinflammation are related to vascular and metabolic risk factors associated with cognitive function [127]. Vascular cognitive impairment, where symptoms arise as a direct consequence of a cerebrovascular event such as stroke or brain haemorrhage, account for approximately 20% of all neurocognitive disorders [128]. Yet vascular factors contribute to the onset and progression in other types of dementia including AD where most dementia cases have mixed pathologies and vascular lesions are a common feature [129]. The biomarkers sections below outline the main results from available RCTs targeting vascular/metabolic factors (Supplementary Tables 5, 6, 7 and 8. Summary of main results in Fig. 2).

### Type-II diabetes interventions

Type-II diabetes (T2D) is a well-established risk factor for dementia [15] and a large body of pre-clinical and clinical evidence supports the notion that these two conditions could share comorbidity and pathophysiological mechanisms [130].

### T2D interventions: associations with neuroimaging biomarkers

So far, studies have mainly focused on investigating the neurodegenerative complications in T2D, through associated neuroimaging markers. Reduced global brain volume and regional atrophy in the hippocampi, basal ganglia, and orbitofrontal and occipital lobes [131] (as well as observed decrease in global grey matter volumes) [132, 133] and subtle white matter microstructural alterations [134] were the most consistent neurodegenerative changes reported, in line with a shared neuropathological aetiology between T2D and dementia. A proof-of-concept RCT testing the effect of intranasal insulin on brain function in older adults with T2D [135] reported increased connectivity between the hippocampal regions and multiple default mode network regions. A significant correlation between resting-state connectivity and cognitive performance was also found, overall suggesting that insulin levels may modulate functional connectivity among brain regions regulating memory and complex cognitive behaviours [135].

Intensive control of blood glucose – with the aim of normalising haemoglobin A1 C (HbA1c) levels among T2D patients- was found to be associated with beneficial effects on imaging biomarkers of neurodegeneration [136]; Compared to standard care, intensive treatment to target HbA1c levels reduced the loss of grey matter volume [136]. Furthermore, reducing HbA1c significantly improved cognition and CBF in the parietal lobe, compared to placebo [137], all in all suggesting potential stabilising effects on neurodegenerative processes.

To date, only one lifestyle intervention aimed at T2D patients has been studied in relation to its potential mechanistic implications for dementia. The US Look AHEAD RCT combined multiple exercise and diet approaches in the context of CVD prevention among > 5000 middle-aged and older type 2 diabetics to track the development of CVD over time [138]. Although the intervention was stopped after a median follow-up of about 10 years, due to no effect on cardiovascular outcomes [138], neuroimaging data reported that the intervention was able to significantly reduce WMH, within the same timeframe [139].

### T2D interventions: associations with ADRD and inflammatory biomarkers

The effect of dysfunctional glucose metabolism on different types of ADRD and neurodegeneration biomarkers and a potentially beneficial effect of T2D treatment on ADRD pathology have been investigated in RCTs. An RCT exploring diet-induced levels of hyperinsulinemia, typical of insulin resistance, were shown to increase CSF levels of neuroinflammatory markers and reduce plasma and CSF levels of Aβ42, thereby potentially increasing the risk of AD [140]. The changes in CSF levels of Aβ42 were associated with levels of CSF neuroinflammatory markers and the thyroid hormone transporter transthyretin. Increased inflammation was modulated by insulin-induced changes in CSF levels of norepinephrine and apolipoprotein E (APOE).

Limited causal evidence related to the effect of pharmacological T2D treatments on specific ADRD pathology/biology mechanisms in cognitively impaired individuals is also available from RCTs. Some interesting lines of evidence have been found for metformin, one of the first line of treatment for T2D. In addition to potential neuro-protective effects (e.g., vascular, metabolic, anti-senescence via genetic modulation) [141–143], it has been proposed that metformin could potentially counteract biological mechanisms of aging, which may also play an important role in neurodegenerative disorders [141]. This suggests that pharmacologic strategies for decreasing insulin resistance and preventing T2D may also help reduce the risk of cognitive impairment. In terms of other pharmacological treatment for T2D, MCI and AD patients treated with rosiglitazone [144], an antidiabetic drug in the class of the thiazolidinedione, stabilized the plasma Aβ42 levels (i.e., unchanged compared to baseline), which are known to decrease with progression of AD [145], compared to the placebo. Similar findings on the plasma Aβ42 levels (including Aβ40/Aβ42) were also reported for treatment with the related drug pioglitazone, in patients with mild AD [137].

### Obesity targeted interventions

Midlife obesity has consistently been associated with an increased risk of dementia. Individuals with a body mass index (BMI) in the obesity range have a 1.4-fold increased risk of dementia [146] whereas a higher BMI during late life has a protective effect [147]. The association between BMI and brain pathology has not been extensively studied in RCTs but is mainly based on observational studies. The impact of weight loss on dementia-related biomarkers has however been studied in relation to other lifestyle interventions, such as diet and exercise (see previous sections).

### Obesity: associations with neuroimaging biomarkers

Observational data link overweight, obesity and higher BMI at midlife with increased risk of AD dementia and higher amyloid burden in the brain later in life [148–151]. Several studies have also found associations between high BMI and decreased brain volumes [152, 153] and cortical thinning [154] later in life. Cross-sectional studies report that obesity is linked with lower FDG-PET in patients with preclinical AD [155] and AD-related grey matter atrophy in people with AD, yet this atrophy pattern did not overlap with Aβ or tau burden [156]. This may point to the notion that high BMI has been observed as a dementia risk factor in midlife yet reversed during late life.

### Obesity: associations with ADRD biomarkers and other fluid biomarkers

The mechanisms linking obesity to atrophy have not been yet fully elucidated. Available evidence suggests that obesity triggers metabolic disturbances [157, 158] such as insulin resistance, high levels of adipokines and cytokines, and advanced glycosylation end products, being linked to higher AD risk [159, 160]. In persons with MCI, intentional weight loss through diet was associated with cognitive improvement. This association was strongest in younger individuals and in APOE-ε4 carriers which emphasizes the importance of early intervention as well as in those with higher dementia risk, as they may benefit more. Also, changes in metabolic homeostasis, i.e., insulin resistance, C-reactive protein, leptin and intake of energy, carbohydrates, and fats were associated with improvement in cognitive tests [161].

Obesity‐associated inflammation has been linked to abnormalities in the blood‐brain barrier, neuroinflammation and neurodegeneration and has been associated with impaired synaptic plasticity and memory [162, 163]. Additionally, as a strong risk factor for hypertension, T2D, and dyslipidaemia, obesity is also an established factor for cerebrovascular risk [164]. As for other risk factors, the relation between obesity and dementia is complex and depend on age and underlying pathology. During late-life, lower BMI or weight loss signifies underlying brain pathology. For example, weight loss in community-dwelling older adults was associated with cognitive decline along with high NfL and low Aβ 42/40 levels [165].

### Anti-hypertensive treatment

Midlife hypertension is a well-known risk factor for dementia [166]. A specific pattern of mid-life hypertension followed by a rapid decrease of blood pressure in late life has been associated with the development of dementia [167]. However, no conclusive evidence is available on whether interventions reducing blood pressure in late life can have an impact on subsequent cognitive impairment/dementia [168]. Additionally, limited evidence is available on potential mechanistic effects linked to dementia for the treatment of hypertension.

### Anti-hypertensive interventions: associations with neuroimaging biomarkers

Observational evidence shows that hypertension is significantly associated with the presence and progression of WMH [169, 170], which, as a highly prevalent MRI marker of cerebral small vessel disease, is linked to both stroke and dementia risk. Furthermore, in a systematic review and meta-analysis including individuals in both mid- and late life, most of the studies included reported a significant association between increased blood pressure level and/or hypertension, and total and/or regional brain volume reduction, with the frontal and temporal lobes being particularly affected [171]. Variability in blood pressure was also associated with similar structural brain changes, e.g., lower brain and hippocampal volumes [172].

Studies comparing different pharmacological therapies for hypertension in absence of a control/placebo showed that effective reduction of high blood pressure can have beneficial effects on regional CBF, both in cognitively normal [173, 174] and AD patients [175]. RCTs evidence also confirms that standard pharmacological treatment of hypertension may have a beneficial impact on neuroimaging markers of neurodegeneration, such as reduced risk of WMH progression and possibly brain atrophy in cognitively normal patients [176].

The effect of intensive pharmacological management of hypertension (systolic blood pressure goal of less than 120 mmHg versus a standard goal of less than 140 mmHg) has also been investigated in relation to the risk of dementia and potential pathological biological mechanisms, within the SPRINT-MIND RCT [177–179]. The primary results of the study showed that intensive blood pressure control significantly reduced the risk of MCI and the combined rate of MCI or probable dementia. Increased trends of annualized change in CBF [180] and smaller increase in WML volume [181] were also reported for the intervention group compared to the standard blood pressure control. When the results of several trials were pooled, patients in the intensive blood pressure control group were reported to have a significantly slower progression of WMH, with the reduction in WMH progression being proportional to the magnitude of the blood pressure control [182].

### Anti-hypertensive interventions: associations with ADRD fluid biomarkers

In a small study, effective anti-hypertensive treatment was found to inhibit CSF angiotensin-converting enzyme activity but however with no effect on CSF Aβ42 [183]. RCTs investigating effects on other fluid biomarkers related to dementia, such as tau and p-tau are currently lacking.

### Hypercholesterolemia treatment

During recent years, consistent reports eg. a meta-analysis including 1.2 million participants [184] have shown an increased incidence of dementia in people with midlife hypercholesterolemia and increased low-density lipoprotein (LDL) levels at midlife is one of the risk factors for dementia [185].

Despite inconclusive data, most observational studies show that the use of statins, originally developed to treat atherosclerosis and cardiovascular disease is associated with neurocognitive disorders [186, 187]. Studies show that treating with statins does not merely result in a vascular risk reduction but could interfere with AD neuropathology and influence other neurodegenerative-related mechanisms such as inflammation [188]. When studying associations with brain-related biomarkers, it may also be important to consider the different solubility properties of statins, as some are lipophilic and thus penetrate more easily the blood–brain-barrier [189].

### Hypercholesterolemia treatment: associations with neuroimaging biomarkers

Neuroimaging biomarkers and their associations with statin use show discrepant results in RCTs. Although statin treatment in older people with increased vascular risk was not associated with change in hippocampal volume [190] patients with atrial fibrillation (on anti-coagulant treatment) receiving statins showed less atrophy in the medial temporal lobe compared to placebo [191]. Since hyper-lipidaemia influences blood flow, one outcome measure to explore statin effects in the brain is by CBF. Based on the available data however, no major effects have been observed; CBF has been shown to be increased in certain brain regions after treatment, while global CBF remained unchanged [192]. Further, high LDL variability has been associated with lower CBF and increased risk of WMH, yet these associations were not either shown to be affected by statin treatment in healthy older people at risk of vascular disease [190]. Conversely, in a trial including older people with hypertension but without dementia, low dose statin treatment combined with anti-hypertensives resulted in a significantly decreased risk of WMH progression and a reduced risk of new-incident vascular lesions compared to only antihypertensive treatment [193]. White matter microstructure and integrity were further shown to be maintained in healthy individuals with a family history of AD undergoing statin treatment [194]. With lower drug dosages, WMH progression was however reduced only in those subjects that already had significant lesions. Another trial show that changes in WMH volume could be predicted by statin treatment yet no group-level differences were seen in WMH between placebo and treatment after 2 years [195]. Furthermore, transient changes in neuronal activity measured by BOLD (Blood Oxygenation Level Dependent) fMRI were seen after statin treatment, without any improved neuropsychological test scores [196].

### Hypercholesterolemia treatment: associations with ADRD biomarkers and other fluid biomarkers

Effects from statin treatment on ADRD biomarkers have shown divergent results: No group differences were seen in CSF Aβ42 or tau between simvastatin treatment and placebo in cognitively normal [197] or pre-dementia stage [198]. When treatment effects between a lipophilic and a hydrophilic statin were compared in healthy hypercholesterolemic subjects, levels of phosphorylated tau were however decreased with the lipophilic statin [199] suggesting that solubility properties of the statins could play a role. Blood biomarkers detecting AD pathology have been investigated in the context of statin use but also here results are inconsistent. In healthy older adults eligible for statins, treatment with the lipophilic statin lovastatin reduced Aβ peptide serum levels in a dose-dependent manner [200]. Studies in other cohorts of healthy older or MCI groups however show that statin use (both lipophilic and hydrophilic) had either no significant effects [201–204] or increased Aβ blood levels [205]. A combined high dose of statin with anti-coagulant treatment in patients diagnosed with atrial fibrillation was shown to reduce levels of several pro-inflammatory proteins in plasma compared to placebo [206] suggesting that inflammation can be one link between statin use and dementia. Further, the inflammatory marker RAGE (Receptor for Advanced Glycation End products) was shown to be reduced by statin treatment [205].

Taken together, the risk reduction strategies targeting metabolic and vascular risk factors have diverse and sometimes complex effects on biomarkers. Biological and clinical heterogeneity within risk groups would be important to take into consideration.

## Social activities

Observational evidence links social participation to reduced risk of dementia. Lifestyle-based interventions often include a social dimension of interaction with other participants during group activities or intervention delivery. In the multimodal FINGER trials, social activity is one of the components [22]. The integration into other domains however adds a complexity of defining and quantifying engagement [207] and biomarker studies related to social activity as single intervention in dementia relevant cohorts are to our knowledge not yet available. It is thought that quality social interactions may support cognitive reserve and brain maintenance by reducing stress and improving cerebrovascular health [208]. In a broader perspective, social activity contributes to mental wellbeing.

### Management of sleep and stress as dementia risk reduction strategies

Previous studies show associations between dementia and factors influencing mental wellbeing such as sleep problems or stress [209, 210]. Additional to social activity, stress management and sleep counselling is included in some of the WW-FINGERS studies (e.g. [211, 212]). For this review we investigated dementia risk reduction RCTs targeting sleep problems or stress as these are novel emerging risk factors and some mechanistic investigations were available (studies listed in Supplementary Table 9, summary of findings in Fig. 2).

### Sleep interventions

Reviews and meta-analyses suggest that sleep disturbances could increase the risk of cognitive impairment or AD [213–216]. A longitudinal cohort study over decades showed that sleep duration was linked to late life burden of Aβ [217]. The potential mechanism is proposed to be a reduced clearance of Aβ by the glymphatic system that is affected by sleep [218]. Despite the evidence, very few RCTs have been conducted testing lifestyle interventions aimed to prevent sleep problems, whilst measuring AD biomarkers.

### Sleep interventions: associations with neuroimaging biomarkers

A physical exercise intervention that included middle-aged obstructive sleep apnea patients resulted in not only improved exercise capacity, but also reduced severity of apnea. The intervention improved also cerebral metabolic glucose rate in the frontal lobe which may have caused the increase in attention/executive functioning, compared to controls [219]. In patients with insomnia and fibromyalgia sleep education was associated with an increase in cortical thickness and a decrease in wake time during the night predicted this change [220]. Sleep therapy for insomnia consisting of bright light therapy, body temperature manipulation, and advice on sleeping habits decreased sleep latency and improved sleep efficiency. Furthermore, during letter and category fluency tests, functional activation in the brain recovered hypoactivation of the medial and inferior prefrontal cortical areas when results were compared with the control group [221].

In addition to lifestyle interventions, a few studies have examined associations with bright or blue light therapy interventions, sleep disturbances, and neurobiological changes [222–224]. Improved sleep quality or quantity was associated with increased volumes of the posterior thalamus, increased thalamo-cortical functional connectivity, and increased axonal integrity of these pathways [223]. Furthermore, decreased functional connectivity was seen in a cluster of regions, which are part of the salience network among young adults exposed to bright light therapy for two weeks. These brain changes were associated with improved sleep quality [224].

### Sleep interventions: associations with BDNF and other fluid biomarkers

Levels of BDNF have been shown to be decreased among people suffering from insomnia and fatigue. In a sleep medicine and/or sleep hygiene intervention sleep disturbances decreased in all groups, yet this did not affect BDNF levels [225]. Improved sleep quality or quantity has however been shown to lower plasma levels of the inflammatory marker IL-6 [222].

The current evidence investigating sleep is limited, but available research in this area suggest sleep problems can be improved with lifestyle interventions or light therapy, and subsequent improvements may have a small yet significant positive effect on brain function and/or structure. Studies were small, and with a relatively short duration. Therefore, the results should be interpreted with caution. Thus, sleep may be seen as a potential component of preventive actions for memory disorders in the future, but the effect of sleep on cognition should be tested with large and long-term RCTs, where qualitatively high standardized methods are used to confirm the results.

### Stress interventions: Mindfulness and meditation

There is increasing evidence for different methods targeting stress management as dementia risk reduction. There is some evidence suggesting that meditation or mindfulness could have positive effects on cognitive performance among older adults [226, 227], but evidence from RCTs and in relation to biomarker outcomes is scarce.

### Mindfulness and meditation: associations with neuroimaging and fluid biomarkers

In the French Age-Well RCT comprised of community-dwelling cognitively unimpaired older adults, a meditation program found some evidence of positive behaviour-related effects, but no intervention effects were observed on brain volumes or perfusion in the anterior cingulate cortex and insula areas [228].

Some exploratory smaller and/or shorter trials have studied the effects of meditation/mindfulness interventions on structural and functional MRI parameters, inflammatory biomarkers, plasma and saliva Aβ, telomere length and activity, and transcription factors regulating aging brain's stress response (REST [229]). Despite some reported positive results (e.g. decreased levels of inflammatory markers and REST) the overall evidence is inconclusive [230–235].

## Dementia risk reduction by multimodal interventions

A priori hypothesis in multimodal interventions is that they affect several pathways and networks. This pleiotropic mode-of-action may therefore require multiple biomarkers to address its effect. Still there is relatively limited biomarker evidence available from the RCTs conducted so far, and the presented results are mixed. This can be due to the heterogeneity of the RCTs, such as differences in target populations and chosen interventions (e.g., content, intensity). Published studies focus mostly on neuroimaging substudies. Effects on e.g. the new AD blood biomarkers [236] have not yet been explored in the context of large, multimodal lifestyle trials. The studies included in this review are listed in Supplementary Table 10 and overall results are summarised in Fig. 2.

### Multimodal interventions: associations with neuroimaging biomarkers

Imaging sub-studies were included in the first three large, long-term multimodal dementia prevention RCTs FINGER, MAPT (a Multimodal Approach for preventing AD) and preDIVA (The Prevention of Dementia by Intensive Vascular care).

The FINGER lifestyle intervention was not associated with changes in brain volumes, cortical thickness, or WMHs over the 2-year core trial period [237]. The observed structural brain changes were overall small in this cohort of at-risk individuals. Baseline status was found to modify the intervention effects to some extent, i.e., cognitive benefits were more pronounced among individuals with higher AD-signature cortical thickness and hippocampal volume [237]. Among FINGER participants with a higher genetic risk score, amyloid deposition assessed by PiB PET increased less in the intervention compared to control group [238]. When diffusion tensor imaging (DTI) was used in attempts to capture earlier, subtle, changes in white matter microstructure, an intervention-related decrease was observed in fractional anisotropy (FA) [239]. This also associated with cognitive improvement. While exploratory, this suggests that a multimodal lifestyle intervention may alter white matter microstructure, and FA could serve as a marker to reflect intervention effects.

The multimodal intervention in MAPT was associated with morphological changes in cortical thickness and subcortical volumetric measures [240]. These changes were also related to better cognitive performance. No intervention-related changes were observed in brain glucose metabolism on FDG-PET [241], or functional connectivity [242]. Amyloid status modified the intervention effects on cognition such that beneficial effects were observed among amyloid-positive, but not amyloid-negative participants [20, 243]. The preDIVA MRI study did not report any intervention-related vascular brain changes [244]. Occurrence of new lacunar infarcts was similar in the intervention and control groups. WMH increased over the 6-year trial period, but similarly in the two groups. WMH load was also studied as a potential modifier of intervention effect in this heterogenous study population, to identify potential subgroups benefitting from the intervention. Individuals with more WMHs (greater vascular burden) tended to benefit more, supporting the rationale of targeting preventive strategies at individuals with risk factors [244].

Imaging findings in smaller/shorter multimodal prevention RCTs have also been mixed. In the MEDEX (Mindfulness, EDucation, and EXercise for Age-Related Cognitive Decline) RCT, which investigated cognitive effects of stress reduction/mindfulness and exercise, no intervention-related changes were found in hippocampal volume or cortical thickness [245]. Intervention-related changes were also not observed in the Project Movimente study (combined exercise and cognitive intervention [246]) or in the Train the Brain study (combined cognitive stimulation, exercise, and music therapy [247]. In the latter study, an intervention-related increase was observed in CBF, particularly in para-hippocampal regions, and fMRI results suggested potentially preserved neural efficiency in the intervention group.

The SUPERBRAIN (South Korean Study to Prevent Cognitive Impairment and Protect Brain Health Through Lifestyle Intervention in At-Risk Elderly People) RCT investigated a FINGER-type, either facility- or home-based multimodal lifestyle program. The study reported positive intervention effects on global cortical thickness (as well as regional cortical thickness of the bilateral frontotemporal lobes, cingulate gyri, and insula), which together with increased serum BDNF, could point towards neuroplastic changes [248, 249]. Yet, changes in white matter integrity (DTI measures) were mostly similar in the intervention and control groups [250]. Positive effects on cortical thickness were also reported in a Japanese RCT (intervention: exercise with cognitive stimulation under dual-task conditions), and these changes correlated with changes in memory [251]. In SUPERBRAIN, some intervention-related changes were also observed in fMRI (changes in regional homogeneity and spontaneous functional activity) [249] and in EEG (changes indicative of increased functional brain networks which also associated with cognitive changes) [252].

A new preprint from the multi-modal intervention study AgeWell.de report that there were no effects on brain imaging markers of neurodegeneration or small vessel disease in the intervention group, yet preliminary findings suggest an association to CBF [253].

### Multimodal interventions: associations with fluid biomarkers

Few studies have investigated the effects of multimodal interventions on fluid AD/dementia-related biomarkers. In FINGER, assessment of plasma metabolomics [254] bioplex inflammatory markers, and AD related blood-based biomarkers (BBM, including 11-year follow-up samples) are currently ongoing. In the smaller neuroimaging subcohort an association were seen between memory improvement and reduced 27-hydroxycholesterol (27-OH), which is an oxysterol linked to CVD and neurodegenerative disorders [255]. Telomere length, which is a marker of ageing and dementia, was found to be maintained by the FINGER intervention, including in ApoE4 carriers [256].

Intervention-related changes in serum BDNF have been reported in some trials (SUPERBRAIN, [248, 249]) but not all (Project Movimente [246]; ENLIGHTEN [111]). In a subcohort of the FINGER RCT intervention group higher baseline levels of the BDNF precursor proBDNF was able to predict memory improvement [257]. In the ENLIGHTEN RCT (combined aerobic exercise and DASH intervention), there were also no changes in peripheral inflammatory markers or VEGF [111]. For VEGF, one intervention study combining exercise and cognitive stimulation reported increasing levels and positive correlations with memory [258].

## Discussion

Lifestyle-based interventions, especially multimodal interventions, may have benefits on decreasing the risk of and/or delaying dementia onset. Considering that up to 45% of the dementia risk can be attributed to modifiable risk factors [15] further developing and tailoring lifestyle modifications could have a significant impact on the number of affected people worldwide. The heterogeneous nature of dementia disorders suggests that multimodal interventions are more likely to be effective in managing the condition. The overall effect size however depends on the intervention intensity, duration and design, as well as target population [259], and thus varies across trials (e.g. in the WW-FINGERS RCTs [18, 212, 260]). Enhancing our understanding of the mechanisms behind modifiable risk factors and lifestyle changes can offer valuable evidence to guide the development of preventive programs. These programs can then be more effectively tailored to address specific risk profiles at both individual and population levels. Knowledge of biosignatures and risk profiles could pave the way toward a more personalized prevention approach (precision prevention). Furthermore, elucidating mechanistic drivers during different modalities in lifestyle interventions may also contribute with clues for novel drug targets and eventually provide an updated multi-mechanistic AD/dementia working model.

As summarized in Fig. 2, the currently available results from RCTs support several potential actions of lifestyle interventions over a variety of pathophysiological mechanisms. Most evidence derives from neuroimaging analyses where some positive effects can be seen on brain structure and functional connectivity for the types of interventions investigated in this review. For ADRD biomarkers there is less available evidence. While some physical exercise interventions have shown positive effects, results for dietary interventions and CVD/metabolic risk reduction are inconsistent. We did not find studies investigating the effects on ADRD markers after cognitive training or multimodal interventions. Trials targeting more exploratory biomarkers are scarce and those available show non-significant findings, except for a steady BDNF increase after physical exercise. Nevertheless, there are limitations and methodological issues in the use of BDNF as a diagnostic biomarker and further validation is needed to ensure its reliability [261]. Overall further evidence is needed to draw conclusions on the molecular benefits of lifestyle interventions in dementia prevention. Although lifestyle interventions have, in several cases, shown cognitive and other related benefits, molecular underpinnings that stimulate resilience to dementia and/or delay its progression are not fully elucidated. In Table 1 we summarize the main challenges with biomarker studies in dementia prevention and suggest alternatives that could facilitate the acquisition of more biomarker measurements in lifestyle-based interventions and advance the field of precision prevention in dementia.

**Table 1:** Challenges and possible approaches for future dementia prevention RCTs

Challenges to be addressed in future trials		Possible approaches
ADRD biomarkers	It is often not feasible to collect cerebrospinal fluid samples in lifestyle-based prevention trials due to costs and/or target groups without substantial impairment requiring diagnostic assessments	The blood-based biomarker (BBM) field is rapidly evolving and BBMs could be applied in large scale RCTs; ⇒ to find the right target groups ⇒ to monitor the intervention ⇒ as outcome of the intervention Alignment of methodology and data sharing (e.g. AD-Workbench)
Neuroimaging biomarkers	Neuroimaging is often conducted in smaller sub-studies with insufficient power to draw conclusions Neuroimaging in dementia risk reduction trials has been focused on classical biomarkers	Optimizing the use of the vast amount of available real-world MRI data collected during regular health care. Portable Low field (LF) MRI may be a more accessible and affordable alternative for conducting neuroimaging in dementia prevention RCTs [262] Alignment of methodology and MRI- data sharing (e.g. TheHiveDB) [263]. Additional markers including markers for brain age/brain reserve
Exploratory biomarkers	Biomarkers for inflammation, vascular markers, oxidative stress, neuroplasticity often differs between RCTs	More focus on analytical and/or clinical validation
Trial design	There is a large heterogeneity in risk reduction/lifestyle-based trials (e.g. considering the target group, type of intervention, exposure time and dose) resulting in sometimes inconclusive results	Prospective harmonization of RCT protocols (e.g. global efforts are currently ongoing in the WW-FINGERS project [22])

### Gaps in knowledge

Biomarker studies in lifestyle RCTs are rarely conducted on a large scale and many are in selected sub-cohorts. There is also a significant variability in biomarker selection across different studies, which presents challenges when making comparisons. While this review focused on traditional biomarkers widely used, there is a growing body of novel exploratory biomarkers that would be potentially useful e.g. in future nutritional interventions [264], however further validation is required. Our literature search showed that physical exercise was the most studied intervention domain, whereas CVD/metabolic interventions had the largest populations (Supplementary Fig. 1). Further, there are significant design differences considering intervention type and duration, participant characteristics and disease/risk stage, making comparisons across studies difficult. Considering that the biology of aging may significantly affect lifestyle risk reduction, where a risk factor at midlife can be protective at older age, markers of biological age could be informative to include in the analyses. Along with age, sex differences in intervention response on biomarkers should be more thoroughly investigated. Protective mechanisms (e.g. autophagy, unfolded protein response, and apoptotic inhibition, reviewed in [265]) are inherently complex and influenced by multiple demographical factors.

Furthermore, most available studies are based on western populations. Whether demographical, socio-economic factors or other individual characteristics may modify intervention effects, including biomarker changes, should be further investigated. Clear opportunities have emerged with the WW-FINGERS project [22] which is testing lifestyle-based multimodal dementia prevention RCTs across diverse demographic and cultural settings (currently 70 countries included). Several studies in low- and middle-income countries (LMICs) are planned or ongoing testing feasibility and efficacy of the multimodal approach [266–269]. The potential of such strategy is reflected by the high prevalence of modifiable risk factors for dementia in LMICs [270]. Regarding fluid biomarkers, an international working group (IWG) gathering experts from the different WW-FINGERS participating countries, is actively working on prospective harmonization within an expanding, global biorepository to ensure high-quality reproducible data collection and joint analyses. Technologies for measuring fluid biomarkers are constantly evolving and worldwide efforts such as the Global Biomarker Standardization Consortium (GBSC) have generated guidelines for measuring fluid biomarkers in dementia. Importantly, recent developments in AD blood biomarkers have improved the accessibility for relevant diagnostic and monitoring tools across the full AD continuum [236] that are easier to implement in non-pharmacological and/or dementia risk reduction RCTs than CSF and neuroimaging biomarkers. Biomarkers that go beyond amyloid and tau pathologies are also important for investigating the complex mechanisms of preventive interventions, yet standardized protocols are still lacking [271].

Targeting younger at-risk populations may be a more efficient approach to reduce disease burden, not only for dementia, but also for other age-related disorders. However, RCTs with very long follow–up times are rarely available. Multimodal interventions, such as those combining cognitive and physical training report long‐term benefits on cognition maintained for up to five years after the training stopped [272] but more long-term follow-ups are needed. With longer follow-ups it would also be possible to assess the sustainability of induced positive effects.

## Future directions and conclusions

Since the pioneering FINGER trial and the launch of the WW-FINGERS project [22], the multimodal model for dementia risk reduction has been developed further towards a precision prevention platform approach. In FINGER 2.0 potential disease-modifying drugs/other compounds are added to a lifestyle intervention, further tailoring to specific risk profiles. The combination of FINGER lifestyle intervention and medical food was tested in the MIND-AD RCT showing good feasibility and adherence in prodromal AD and signs of potential cognitive benefits [273]. The MET-FINGER is the first trial testing a full FINGER 2.0 model combining lifestyle intervention and metformin treatment in older healthy adults at risk of dementia and with indicators of increased risk of diabetes (currently ongoing in the UK, Finland, and Sweden) [274]. As new treatments and disease-modifying drugs are progressing in the drug development pipeline the personalised and disease-mechanistic approach will be further refined. Future adaptations could include a flexible RCT platform design, where the FINGER lifestyle model will be combined with different pharmacological putative disease-modifying treatments in different individuals/sub-population, depending on their specific risk profile [21].

Future combination treatments may be more efficient as complementary mechanisms could be targeted simultaneously. In a complex, multifactorial disease such as AD a multi-mechanistic approach has the potential to be more clinically beneficial and may also reduce side-effects from disease-modifying drugs. How brain aging mechanisms interact with ADRD biomarkers and lifestyle changes also remains to be determined. In this new era of AD research, with novel disease-modifying therapies in the horizon and knowledge about the impact of modifiable risk factors constantly increasing, there is renewed hope for tools and opportunities to delay, prevent, or potentially cure the disease. Identifying personalized tools is the next step for an efficient and adaptive prevention approach in dementia where a deeper knowledge on mechanisms and biomarkers is essential.

## Supplementary Information

Supplementary Material 1: Table S1. Search terms for literature identification Supplementary table 2. Meta-analyses and randomized controlled trials on the effect of physical exercise interventions on dementia related biomarkers. Supplementary table 3. Randomized controlled trials on the effect of cognitive intervention on dementia related biomarkers. Supplementary table 4. Randomized controlled trials on the effect of dietary intervention on dementia related biomarkers [275]. Supplementary table 5. Randomized controlled trials on the effect of diabetes type 2 dementia related biomarkers. Supplementary table 6. Randomized controlled trial on the effect of obesity intervention onintervention on dementia related biomarkers. Supplementary Table 7. Randomized controlled trials on the effect of hypertension interventions on dementia related biomarkers. Supplementary Table 8. Randomized controlled trials on the effect of hypercholesterolemia interventions oninterventions on dementia related biomarkers. Supplementary Table 9. Randomized controlled trials on the effect of sleep or stress-reducing interventions on dementia related biomarkers. Supplementary Table 10. Randomized controlled trials on the effect of multimodal interventions on dementia related biomarkers.

Supplementary Material 2: Supplementary Figure 1. Overview of the number of randomized controlled trials conducted for the different lifestyle intervention domains included in this reviewand total numbers of participants included in these

## Data Availability

Not applicable.

## Abbreviations

18-FDG-PET: 18-Fluorodeoxyglucose positron emission tomography
27-OH: 27-Hydroxycholesterol
AD: Alzheimer’s disease
ADRDs: Alzheimer’s Disease Related Dementias
Aβ: Amyloid β peptide
APOE: Apolipoprotein E
BMI: Body mass index
BDNF: Brain derived neurotrophic factor
CVD: Cardiovascular disease
CNS: Central nervous system
CBF: Cerebral blood flow
CSF: Cerebrospinal fluid
DASH: Dietary Approaches to Stop Hypertension
DTI: Diffusion tensor imaging
DMTs: Disease modifying therapies
FINGER: Finnish Geriatric Intervention Study to Prevent Cognitive Impairment and Disability
FA: Fractional anisotropy
HbA1c: Haemoglobin A1C
IL: Interleukin
LD: Lipid-depleted amyloid
LDL: Low density lipoprotein
MRI: Magnetic resonance imaging
MAPT: A Multimodal Approach for preventing AD
MEDEX: Mindfulness, EDucation, and EXercise for Age-Related Cognitive Decline
MeDi: Mediterranean diet
MCI: Mild cognitive impairment
MIND-diet: Mediterranean-DASH Intervention for Neurodegenerative Delay
Neurofilament light chain: NfL, a marker of axonal damage
PreDIVA: The Prevention of Dementia by Intensive Vascular care
RAGE: Receptor for Advanced Glycation End products
RCT: Randomized clinical trial
SUPERBRAIN: South Korean Study to Prevent Cognitive Impairment and Protect Brain Health Through Lifestyle Intervention in At-Risk Elderly People
REST: Transcription factors regulating aging brain's stress response
t-tau: Total tau
T2D: Type-II diabetes
TNF-α: Tumor necrosis factor α
VEGF: Vascular endothelial growth factor
WMH: White matter hyperintensities
WW-FINGERS: World-Wide FINGERS
