# Supplementary material for: Mechanisms of interventions targeting modifiable factors for dementia risk reduction
Source: Mol Neurodegener. 2025 Jun 23;20:75. doi: 10.1186/s13024-025-00845-w (PMC12186355; doi:10.1186/s13024-025-00845-w)
Supplement: Supplementary file 1 — Supplementary Material 1: Table S1. Search terms for literature identification Supplementary table 2. Meta-analyses and randomized controlled trials on the effect of physical exercise interventions on dementia related biomarkers. Supplementary table 3. Randomized controlled trials on the effect of cognitive intervention on dementia related biomarkers. Supplementary table 4. Randomized controlled trials on the effect of dietary intervention on dementia related biomarkers [275]. Supplementary table 5. Randomized controlled trials on the effect of diabetes type 2 dementia related biomarkers. Supplementary table 6. Randomized controlled trial on the effect of obesity intervention onintervention on dementia related biomarkers. Supplementary Table 7. Randomized controlled trials on the effect of hypertension interventions on dementia related biomarkers. Supplementary Table 8. Randomized controlled trials on the effect of hypercholesterolemia interventions oninterventions on dementia related biomarkers. Supplementary Table 9. Randomized controlled trials on the effect of sleep or stress-reducing interventions on dementia related biomarkers. Supplementary Table 10. Randomized controlled trials on the effect of multimodal interventions on dementia related biomarkers. [file 13024_2025_845_MOESM1_ESM.docx]

**Table S1. Search terms for literature identification**

| **Paragraph in paper** | **Search terms** |
| --- | --- |
| Physical Exercise | (“physical activity” OR “walking” OR “exercise” OR “physical exercise" OR “exercise training” OR “exercise therapy” OR “aerobic exercise” OR “aerobic training” OR “interval training” OR “resistance exercise” OR “resistance training” OR “combined exercise” OR “concurrent training” OR “concurrent exercise” OR “isometric exercise")  AND (biofluid OR blood OR plasma OR serum OR imaging OR MRI OR “magnetic resonance imaging” OR PET OR “positron emission tomography” OR CSF OR “cerebrospinal fluid”) AND biomarker* OR biosignature* OR signature* AND (cognition OR cognitive OR alzheimer* OR dementia) |
| Cognitive training | ("neurogenesis" OR "synaptic plasticity" OR "synaptogenesis" OR "hippocampal volume" OR "brain volume" OR "cognitive training" OR "cognitive stimulation" OR "brain plasticity" OR "neuronal pentraxins" OR "long-term potentiation" OR "long-term depression" OR "meta plasticity" OR "synaptic efficacy" OR "cognitive reserve" OR "functional networks" OR "synapse connectivity" OR "neuronal networks" OR "functional connectivity" OR "neurotrophic factors" OR "synaptic density" OR "cognitive resilience") AND (biofluid OR blood OR plasma OR serum OR imaging OR MRI OR “magnetic resonance imaging” OR PET OR “positron emission tomography” OR CSF OR “cerebrospinal fluid”) AND biomarker* OR biosignature* OR signature* OR "EEG" OR "BOLD" OR "MEG" AND (cognition OR cognitive OR alzheimer* OR dementia) |
| Diet | (diet*[TITLE/ABSTRACT] NOT (Supplement* [TITLE]) AND (biofluid OR blood OR plasma OR serum OR imaging OR MRI OR "magnetic resonance imaging" OR PET OR "positron emission tomography" OR CSF OR "cerebrospinal fluid" OR biomarker* OR biosignature* OR EEG OR BOLD OR MEG) AND (cognition OR cognitive OR Alzheimer OR dementia OR brain) |
| Monitoring of Cardiovascular/metabolic risk factors | ("diabetes" OR "insulin resistance" OR "hyperglycemia" OR "hypoglycemic agent*" OR "hypoglycemic treatment*" OR "hypoglycemic drug*" OR "hypoglycemic medication*" OR "antiglycemic agent*" OR "antiglycemic treatment*" OR "antiglycemic drug*" OR "antiglycemic medication*" OR "glucose-lowering agent*" OR "glucose-lowering drug*" OR "glucose-lowering treatment*" OR "glucose-lowering medication*" OR "glucose-lowering intervention*") AND (biofluid OR blood OR plasma OR serum OR imaging OR MRI OR “magnetic resonance imaging” OR PET OR “positron emission tomography” OR CSF OR “cerebrospinal fluid”) AND biomarker* OR biosignature* OR signature* AND (cognition OR cognitive OR alzheimer* OR dementia) |
|  | ("overweight" OR “obesity" OR "body weight" OR "body mass index" OR "weight loss" OR "body weight changes" OR "weight reduction" OR "weight-loss medication*" OR "weight-loss treatment" OR "weight-loss drugs" OR "bariatric surgery" OR "gastric bypass surgery") AND (biofluid OR blood OR plasma OR serum OR imaging OR MRI OR “magnetic resonance imaging” OR PET OR “positron emission tomography” OR CSF OR “cerebrospinal fluid”) AND biomarker* OR biosignature* OR signature* AND (cognition OR cognitive OR alzheimer* OR dementia) |
|  | ("blood pressure" OR "systolic" OR "diastolic" OR "hypertension" OR "prehypertension" OR "anti-hypertensive" OR "antihypertensive") AND (biofluid OR blood OR plasma OR serum OR imaging OR MRI OR “magnetic resonance imaging” OR PET OR “positron emission tomography” OR CSF OR “cerebrospinal fluid”) AND biomarker* OR biosignature* OR signature* AND (cognition OR cognitive OR alzheimer* OR dementia) |
|  | (“cholesterol” OR “hypercholesterolemia” OR “dyslipidemia” OR “HDL-cholesterol” OR “LDL-cholesterol” OR “triglycerides” OR “cholesterol-lowering agent*” OR “cholesterol-lowering drug*” OR “cholesterol-lowering treatment*” OR “cholesterol-lowering medication*” OR “cholesterol-lowering intervention*” OR “anti-cholesterol agent*” OR “anti-cholesterol drug*” OR “anti-cholesterol treatment*” OR “anti-cholesterol medication*” OR “anti-cholesterol intervention*” OR “statins”) AND (biofluid OR blood OR plasma OR serum OR imaging OR MRI OR “magnetic resonance imaging” OR PET OR “positron emission tomography” OR CSF OR “cerebrospinal fluid”) AND biomarker* OR biosignature* OR signature* AND (cognition OR cognitive OR alzheimer* OR dementia) |
| Psychosocial stimulation | ("Sleep" OR "Insomnia" OR “Mindfulness”) AND (biofluid OR blood OR plasma OR serum OR imaging OR MRI OR “magnetic resonance imaging” OR PET OR “positron emission tomography” OR CSF OR “cerebrospinal fluid”) AND biomarker* OR biosignature* OR signature* AND (cognition OR cognitive OR alzheimer* OR dementia) |

| **Supplementary table 2.** Meta-analyses and randomized controlled trials on the effect of physical exercise interventions on dementia related biomarkers | | | | | |
| --- | --- | --- | --- | --- | --- |
| **Study** | **Design** | **Population** | **Intervention** | **Biomarker outcome** | **Findings** |
| **Meta-analyses with neuroimaging as outcome** | | | | | |
| Ji et al. 2021 (271) | 22 RCTs | N=2670 healthy older adults and persons with MCI  Mean age >60 years, sex not specified | Exercise of various modalities,  6 weeks-2 years  Frequency not specified | -structural MRI  -DTI  -fMRI | Physical exercise associated with in structural and functional changes associated with cognitive changes |
| Hvid et al. 2021 (27) | 7 RCTs | N=841 healthy older adults  Mean age >65 years, both sexes | Exercise of various modalities,  3-12 months  2-5 sessions/week | Hippocampus volume | No statistically significant effect of long-term exercise |
| Hvid et al. 2021 (27) | 3 RCTs | N=256 individuals with MCI  Age=55-87 years, both sexes | Exercise of various modalities,  6-12 months  1-3 sessions/week | Hippocampus volume | No statistically significant effect of long-term exercise |
| Gogniat et al. 2021 (28) | 14 RCTs | N=1185 healthy older adults and individuals with MCI  Mean age=70 years, both sexes | Exercise of various modalities,  Total intervention duration 18-374 hours,  0.5-1.5 hours/session  1-3 sessions/week | Brain volume | No statistically significant effect of long-term exercise on overall brain volume outcomes |
| Zheng et al. 2019 (35) | 9 RCTs | N=412 healthy older adults  Age >55 years, sex not specified | Exercise of various modalities  3-24 months  1-5 sessions/week | fMRI | Regional increases/activation for exercise intervention compared to controls |
| **RCTs with neuroimaging as outcome (not included in the meta-analyses above)** | | | | | |
| Pani et al. 2022 (23) | RCT | N=105 healthy older adults  Age=70-77 years, 49.5% female | 60 months of moderate-intensity continuous training (MICT) or high-intensity interval training (HIIT),  2 sessions/week  50 minutes/session for continuous training or 4x4 minute intervals for high-intensity training | Brain volumes | No difference in structural complexity between groups. A positive association between cardiorespiratory fitness and structural complexity of the cerebral gray matter was however seen. |
| Soshi et al. 2021 (24) | RCT | N=47 healthy older adults  Age >60 years, (range 61-82), 46% female | 3 months of multicomponent exercise,  1 session/week  90 minutes/session | Brain structure and function | Increased gray matter volume and thickness in several prefrontal areas in the intervention group and reduced hippocampal gray matter volume in the control group |
| Colmenares et al. 2021 (25) | RCT | N=94 healthy older adults  Age=60-80 years, 68% female | 24 weeks of dance training  3 sessions/week  60 minutes/session | White matter integrity | There were significant effects of the intervention on total white matter and the genu of the corpus callosum |
| Broadhouse et al. 2020 (26) | RCT | N=43 older adults with MCI  Age >55 years (mean 69.5), 69% female | 6 months of high-intensity resistance training  2-3 sessions/week, each session lasting 90 minutes | Secondary:  Hippocampal subfields and posterior cingulate (PC) structural and functional trajectories | Significant long-term effects for protecting participants from 2% to 3% volumetric loss in Alzheimer-vulnerable hippocampal subfields |
| Sinclair et al. 2021 (29) | RCT | N=108 older adults at risk for dementia  Age >60 years | 24 months of home-based moderate-intensity aerobic physical exercise,  >150 minutes/week | Global and regional brain atrophy | There was no significant effect of the intervention on total grey matter volume or total white matter volume |
| Voss et al. 2013 (31) | RCT | N=70 healthy older adults  Age= 55-80 years, (mean 65), 64.3% female | 12 months of moderate-intensity walking  3 sessions/week  40 minutes/session | White matter integrity | The intervention did not significantly affect white matter integrity |
| Arild et al. 2022 (32) | RCT | N=105 healthy older adults  Age=70-77 years, 49.5% female | 60 months of moderate-intensity continuous training (MICT) or high-intensity interval training (HIIT),  2 sessions/week  50 minutes/session for continuous training or 4x4 minute intervals for high-intensity training | White matter hyperintensities | There were no significant effects of either MICT or HIIT in terms of attenuation white matter hyperintensities growth |
| Venkatraman et al. 2020 (33) | RCT | N=98 older adults with MCI or subjective memory complaints  Age >60 years, (median 73), 56% female | 24 months of home-based moderate-intensity aerobic physical exercise,  >150 minutes/week | White matter hyperintensities and hippocampal volume loss | There was no significant effect of the intervention |
| Pani et al. 2022 (34) | RCT | N=105 healthy older adults  Age=70-77 years, 49.5% female | 60 months of moderate-intensity continuous training (MICT) or high-intensity interval training (HIIT),  2 sessions/week  50 minutes/session for continuous training or 4x4 minute intervals for high-intensity training | Structural brain complexity, measured as cortical thickness and volume, and fractal dimension of the cortical lobes, cerebral and cerebrally gray and white matter | There were no significant effects of either MICT or HIIT on structural brain complexity |
| Liu et al. 2019 (36) | RCT | N= 62  Age =50-70 years | Tai Chi Chuan, Baduanjin for 60 min per session, 5 times per week for 12 weeks. Every session included a sequence of 10 min of warm-up, 30 min of exercises, 10 min of breathing techniques and 10 min of relaxation. | Resting-state functional connectivity | Tai Chi Chuan increased resting-state functional connectivity while Baduanjin decreased resting-state functional connectivity and  both the Tai Chi Chuan and Baduanjin groups demonstrated increased cognition |
| **RCTs with PET as outcome** | | | | | |
| Robinson et al. 2018 (41) | RCT | N=27 healthy young and old individuals  Age 18 to 30 (young) and 65-80 (old), 59.3% female | 3 months of high-intensity aerobic exercise,  3 sessions/week  4x4 minute intervals | Brain glucose uptake during rest | There were significant effects of the intervention for increasing brain glucose uptake |
| Shimada et al. 2017 (43) | RCT | N=25 older women  Age 75-83 (mean 84) | 3 months of multicomponent exercise,  2 sessions/week  90 minutes/session | Brain glucose uptake during walking | There was no significant effect (p>0.05) of the intervention for increasing brain glucose uptake during walking. |
| Vidoni et al. 2021 (45) | RCT | N=117 older adults without cognitive impairment but with elevated, sub-threshold levels of cerebral amyloid  Age >65 years, 67.5% female | 52 weeks of moderate-intensity aerobic exercise,  3-5 sessions/week,  maximum 50 minutes/sessions | Cerebral amyloid in terms of 18F-AV45 standard uptake value ratio (SUVR), whole brain volume and hippocampal volume | No significant effects of exercise on the primary outcome of global amyloid burden or whole brain and hippocampal volume |
| **RCTs with cerebral blood flow and oxygenation as outcome** | | | | | |
| Thomas et al. 2020 (37) | RCT | N=30 individuals with MCI  Age= 55-80 years, 47% female | 12 months of moderate-to-vigorous-intensity aerobic exercise,  3-5 sessions/week  25-40 minutes/session | Cerebral blood flow and brain volume | There were significant effects of the intervention for increasing cerebral flood flow in the anterior cingulate cortex (p= 0.016), and decreasing cerebral flood flow of the posterior cingulate cortex (p=0.01). There were no significant effects on brain volume (p>0.05). |
| Tomoto et al. 2021 (38) | RCT | N=52 individuals with MCI  Age= 55-80 years, 54% female | 12 months of moderate-to-vigorous-intensity aerobic exercise,  3-5 sessions/week  25-40 minutes/session | Carotid arterial stiffness and cerebral blood flow | There were significant effects of the intervention for decreasing carotid β-stiffness index and cerebral blood flow velocity pulsatility. |
| Green et al. 2021 (39) | RCT | N=63 healthy adults  Age >50 years, 73% female | 6 months of either moderate-intensity land walking or water walking,  3 sessions/week  15-50 minutes/session | Cerebrovascular functions | Intracranial blood flow velocities (middle cerebral artery and posterior cerebral artery)  Exercise may induce modest changes in autoregulation and CO2 reactivity. |
| Coetsee et al. 2017 (40) | RCT | N= 67  Age= 55-75 years | Participants were randomized into a resistance training group, high-intensity interval training group, moderate continuous training group. | Cerebral oxygenation during the Stroop task | (a) 16 weeks of exercise training resulted in more efficient cerebral oxygenation during cortical activation compared to a no-exercise control group; (b) high-intensity interval training and moderate continuous aerobic training are superior to resistance training for task-efficient cerebral oxygenation and improved oxygen utilization during cortical activation in older individuals |
| **Meta-analyses with BDNF and other fluid biomarkers as outcome** | | | | | |
| Yu et al. 2021 (44) | Meta-analysis of 3 RCTs | N=221 older adults with MCI  Age and sex not specified | Long-term Baduanjin exercise (fitness qigong),  3-6 months  1 sessions/week | Tau protein and Aβ_1_**_–_**_42_ in the cerebrospinal fluid | A statistically significant effect on lowering Tau protein content in favor of Baduajin exercise (weighted mean difference = –166.69, 95% CI, –196.93 to –136.45; *P<*0.00001), but a negative effect whereby Aβ_1_**_–_**_42_ content increased (weighted mean difference = 180.39, 95% CI, 134.24 to 226.55; *P <* 0.00001). |
| Ma et al. 2022 (52) | 7 RCTs | N=310 older adults with MCI  Age=64-83 years, both sexes | Long-term exercise of different modalities,  12-28 weeks  2-3 sessions/week | BDNF (primary)  IGF-1, VEGF, and FDF-2 (secondary). | A statistically significant effect on BDNF in favor of long-term exercise (SMD: 0.31, 95% CI, 0.08 to 0.54, p=0.009).  Regarding secondary outcomes, there were no statistically significant effects on VEGF (SMD: 0.23, 95% CI, -0.16 to 0.61, p=0.25) or FGF-2 (SMD: 0.04, 95% CI, -0.35 to 0.42, p=0.86), but there was a statistically significant effect on IGF-1 in favor of exercise (SMD: 0.42, 95% CI, 0.03 to 0.81, p=0.03). |
| Ma et al. 2022 (52) | 6 RCTs | N=266 older adults with MCI  Age= 64-83 years, both sexes | See above | TNF-α  IL-1β, IL-6, IL-10, CRP, and IFN- γ | A statistically significant effect of long-term exercise on TNF-α in favor of long-term exercise. Regarding secondary outcomes, there were no statistically significant effects except for CRP in favoring exercise |
| Fernández-Rodríguez et al. 2021 (60) | 4 RCTs | N=129 healthy young adults  Mean age >60 years, both sexes | Acute high-intensity aerobic exercise,  Duration 7-60 minutes | BDNF | A statistically significant effect favoring acute high-intensity exercise (pooled ES: 0.55, 95% CI, 0.12 to 0.98). |
| Fleitas et al. 2022 (65) | 10 RCTs | N=597 sedentary older adults without cognitive impairment  Age >60 years, both sexes | Long-term aerobic or resistance exercise,  4-48 weeks  2-5 sessions/week | BDNF | No statistically significant effect in the primary analysis of long-term aerobic or resistance exercise (SMD: 0.06 (95% CI, -0.10 to 0.22, p=0.47)  The results appeared similar in subgroups where aerobic and resistance exercise interventions were analyzed separately. |
| Wang et al. 2022 (68) | 21 RCTs | N=809 healthy adults  Mixed age range, both sexes | Acute exercise in 5 trials, Duration 5-50 minutes  Long-term exercise in 17 trials,  4-24 weeks  2-7 sessions/week | BDNF | A statistically significant effect favoring both acute exercise (SMD: 1.20, 95% CI, 0.36 to 2.04, p=0.005) and long-term exercise (SMD: 0.68, 95% CI, 0.27 to 1.08, p=0.001).  Subgroup analyses suggested more pronounced effects of long-term exercise in females, individuals aged >60 years, and from aerobic exercise specifically. |
| Ruiz-González et al. 2021 (72) | 4 RCTs | N=135 older adults with MCI  Age=68 -70 years, both sexes | Long-term exercise of different modalities,  8-26 weeks  3-4 sessions/week | BDNF | No statistically significant effect in favor of long-term exercise (SMD: 1.07, 95% CI, -0.14 to 2.28, p=0.08) |
| Marinus et al. 2019 (71) | 11 RCTs | N=464 general older adults without cognitive impairment (except in 2 of the trials)  Age >60 years, both sexes | Long-term aerobic or strength exercise,  6-24 weeks  2-3 sessions/week | BDNF | A statistically significant effect favoring long-term aerobic or strength training (SMD: 0.53, 95% CI, 0.31 to 0.75) |
| **RCTs with BDNF and other biomarkers as outcome (not included in the meta-analyses above)** | | | | | |
| Rodziewicz-Flis et al. 2022 (49) | RCT | N=35 older women without cognitive impairment  Age=65-82 years | 12 weeks of moderate-intensity dance training or balance training  Both interventions were performed 3 times/week, each lasting 50 minutes | Amyloid precursor protein and serotonin concentrations | Significant within-group effects of both dance and balance training on increasing the concentration of amyloid precursor protein and decreasing the concentration of serotonin with the only significant between-group difference being serotonin concentration between balance training and control |
| Behrendt et al. 2021 (61) | RCT | N=38 healthy older adults  Age 55-75 years, sex not specified | 12 weeks of either badminton or cycling,  1 session/week  50 minutes/session | BDNF, IGF-1, IL-6 assessed acutely after one session and after the 12-week intervention | Significant effects of both interventions for acutely increasing serum BDNF, IGF-1, and IL-6. In terms of chronic effects, there were no significant differences between the interventions and the control group. |
| Li et al. 2021 (62) | RCT | N= 29  Age= 64.8 years | HIIT group (4 × 3 min at 90% VO2max interspersed with 3 min at 60% VO2max) and a vigorous-intensity continuous training group (VICT) (25 min at 70% VO2max) and submitted to 12 weeks of training. | Serum neurotransmitters and neurotrophic factors were determined at baseline and post training. | Serum concentrations of BDNF, nerve growth factor, neurotrophin-3 and neurotrophin-4 increased significantly in the HIIT and VICT groups after training. Compared to VICT, HIIT significantly increased the serum neurotrophin-3 concentration. Serum concentrations of the neurotransmitters acetylcholine, dopamine and serotonin trended upward with training |
| Cha et al. 2022 (63) | RCT | N= 20  Mean age=74.80 years | Square-stepping exercise program for 70 min per session, twice a week, for 12 weeks with a certified instructor | BDNF | Significant improvement in BDNF levels for intervention group and a significant decrease in the BDNF level in the control group |
| Devenney et al. 2019 (66) | RCT | N=64 individuals with MCI  Mean age=70.5, 53% female | Acute bout of high-intensity aerobic exercise, graded cardiopulmonary exercise test to volitional exhaustion | BDNF | There was a significant effect of an acute bout of high-intensity exercise for increasing BDNF concentration |
| Tsai et al 2018 (67) | RCT | N=66 older adults with MCI  Age=60-80 years, 57.6% female | Acute bout of aerobic or resistance exercise, 30 minutes of moderate-intensity cycling on a cycle ergometer, or 30 minutes of moderate-intensity machine- and free-weight-based resistance training | BDNF, IGF-1, VEGF, FGF-2 | Levels of BDNF and IGF-1 increased significantly from baseline to 5 minutes after an acute bout of aerobic exercise, with similar results for IGF-1 after an acute bout of resistance exercise, but the levels of these markers approached baseline levels 20 minutes later. There were no changes in FGF-2. |
| Segal et al. 2012 (69) | RCT | N=54 healthy older adults and older adults with MCI  Mean age=69 (healthy) and 71 years (MCI), 59.3% female | Acute both of aerobic exercise,  6 minutes of moderate intensity cycling on a cycle ergometer. | Endogenous noradrenergic response, salivary alpha-amylase (sAA) | There was a significant effect of the intervention for increasing acute levels of endogenous norepinephrine in both individuals with and without MCI. |
| Shaaban et al. 2019 (70) | RCT | N=24 older adults without cognitive impairment  Age=70-89, 83% female | 24 months of multicomponent exercise,  2-6 sessions/week | Cerebral small vein integrity, BDNF, VEGF | There was a significant effect of the intervention for increasing straight vein length. There were no significant effects on tortuous length or tortuosity ratio, and no effects on BDNF or VEGF |
| Vaughan et al. 2014 (64) | RCT | N=49 older women without cognitive impairment  Age=65-75 years | 16 weeks of multimodal moderate-intensity exercise,  2 sessions/week  60 minutes/session | BDNF | There was a significant effect of the intervention for increasing peripheral BDNF concentration |
| Coelho-Júnior et al. 2020 (73) | RCT | N=45 healthy older women  Age >60 years | 22 weeks of moderate-to-vigorous-intensity traditional resistance training or combined traditional resistance- and power training,  2 sessions/week | BDNF | There were no significant effects of any of the training protocols on BDNF concentration |
| Zlibinaite et al. 2021 (74) | RCT | N=63 healthy adults  Age=38-56 years | 60-minute of moderate-intensity cycling sessions for 8 weeks: 40 sessions in total. | BDNF | The intervention had no effect on BDNF levels |
| Marston et al. 2019 (75) | RCT | N=45 healthy adults  Age=41-69 years, 12% female | 12 weeks of moderate-intensity or high-intensity resistance training,  2 sessions/week | BDNF | There were no significant effects of the interventions on BDNF, IGF-1, VEGF, or homocysteine |
| Fragala et al. 2014 (76) | RCT | N=25 healthy older adults  Age >60 years, (mean 70.6), sex not specified | 6 weeks of moderate-intensity resistance training,  2 sessions/week | BDNF | The intervention had no effects on BDNF concentration, with clinical inferences indicating “very unlikely beneficial” effects. |

| **Supplementary table 3.** Randomized controlled trials on the effect of cognitive intervention on dementia related biomarkers | | | | | |
| --- | --- | --- | --- | --- | --- |
| **Study** | **Design** | **Population** | **Intervention** | **Biomarker outcome** | **Findings** |
| **RCTs with neuroimaging as outcome** | | | | | |
| Engvig et al. 2012 (105) | RCT | N=41 participants from the general population  Mean age = 61 years | Cognitive training (memory training) for 10 weeks | White matter microstructure | Cognitive training group showed a relative increase in fractional anisotropy compared with controls |
| Gallen et al. 2016 (81) | RCT | N= 20 cognitively normal older adults  Mean age = 64.40 years | Strategic Memory and Reasoning Training for 12 weeks | Functional connectivity (as measured by resting-state fMRI) | More segregated/modulated brain networks exhibited greater improvements in the cognitive training group |
| Anderson et al. 2014 (83) | RCT | 62 older adults  Age=55-70 years | Auditory based cognitive training for 8 weeks | Measures of neural electrophysiology | The benefits of short-term auditory-based cognitive training for older adults, specifically in terms of improved response peak timing to speech in noise and enhanced processing speed, were sustained six months after the training ended. |
| Ciarmiello et al. 2015 (84) | RCT | N=30 older adults with amnestic mild cognitive impairment  Mean age= 71 years | Neuropsychological cognitive training (stimulate attention, execution function, short-term and long-term memory) for 8 weeks. | Metabolic activity on FDG-PET | Significant association between brain metabolic activity and cognitive function was found in the cognitive training group, relative to controls. |
| Deng et al. 2019 (85) | RCT | N=50 older adults  Mean age=72 years | Cognitive training (either multi-domain or single domain). Multi-domain cognitive training group targeting memory, reasoning, problem-solving strategies, visual-spatial map reading skills, handicrafts, and physical exercise.  The single-domain training targeting reasoning skills for 12 weeks. | Functional connectivity on resting-state fMRI | Local functional connectivity integration in the brains of older adults can be improved through cognitive training.  Older participants who underwent cognitive training showed more integrated local functional connectivity after training compared to those in the control group. |
| Engvig et al. 2014 (86) | RCT | N=61 older adults with subjective memory impairment  Mean age = 60.90 years | Episodic memory cognitive training for 8 weeks | Gray matter volume | Cognitive training related brain changes were found.  Differences in left hippocampal volume change were identified among participants with subjective memory impairment. These brain changes were related to verbal recall improvement. |
| Chen et al. 2021 (87) | RCT | N=84 older adults with amnestic mild cognitive impairment  Age=60-90 years. | Vision-based speed of processing training and active control (mental leisure activities) for 6 w | Structural brain networks on diffusion tensor imaging | Enhanced structural segregation were found in some brain networks – implying neuroplasticity.  Learners (those in the cognitive training group) showed significantly greater global clustering coefficients after intervention, compared to controls. |
| Chen et al. 2020 (88) | RCT | N=84 older adults with amnestic mild cognitive impairment  Age=60-90 years | Vision‐based speed of processing training oriented cognitive training for 6 weeks. | Task‐based fMRI activity | ECG derived autonomic nervous system segment was associated with learning and significantly predicted training‐induced neuroplasticity in the dorsal anterior cingulate cortex and select frontal regions during task fMRI. |
| Berry et al. 2010 (89) | RCT | N=30 healthy older adults  Mean age=71.93 years | Cognitive training (10 hours of visual cognitive training using the Sweep Seeker program) for 3–5 weeks. | EEG‐derived measures of event-related potentials. | Perceptual discrimination training can directly improve working memory in older adults, and that training designed to enhance perceptual abilities can also benefit untrained cognitive functions, such as working memory. |
| Chapman et al. 2015 (90) | RCT | N=37 cognitively normal older adults  Mean age=62.90 years | Gist reasoning training for 12 weeks | Cerebral blood flow measured using pseudo-continuous arterial spin labeling MRI; functional connectivity assessed using resting-state fMRI; and white matter integrity measured using diffusion tensor imaging. | Cognitive training in older adults lead to significant improvements in brain function, connectivity, and structure. Specifically, the training increased cerebral blood flow, enhanced connectivity within key brain networks, and improved white matter integrity. |
| Mishra et al. 2015 (91) | RCT | N=32 older adults  Mean age=71.93 years | Perceptual training for 3-5 weeks | EEG measures of event-related potential recordings | Perceptual training improved ability to discriminate challenging visual tasks and enhanced working memory linked to changes in brain activity suggesting that the training helps older adults better focus their attention on quickly presented stimuli. |
| Motes et al. 2018 (92) | RCT | N=57 cognitively normal older adults  Mean age=63.20 years | Higher order cognitive training for 12 weeks | Task-related fMRI signals; reaction-time-related fMRI signals; pseudo-continuous arterial spin labeling to measure cerebral blood flow; and measures of cerebrovascular reactivity | Higher-order cognitive training improved processing speed and altered brain activity patterns in older adults. |
| Elcombe et al. 2014 (94) | RCT | N=34 adults at-risk of cognitive decline  Mean age=66.8 years | Combined Healthy Brain Ageing psychoeducation and cognitive training program for 8 weeks | Hippocampal volume | Increases in hippocampal volume over an 8-week period were linked to higher cognitive reserve.  Conversely, decreases in hippocampal volume were associated with depression and disability.  Cognitive training did not affect hippocampal size, suggesting that cognitive reserve, cognitive functioning, and depression are more critical factors influencing hippocampal volume in at-risk older adults, compared to cognitive training. |
| Suárez-Méndez et al. 2022 (95) | RCT | N=90 healthy adults and control group with subjective cognitive decline  Age= 60-80 years | Cognitive training for cognition and daily performance for 10 weeks | Functional connectivity | After a 10-week cognitive training program, participants with subjective cognitive decline and healthy controls showed reduced abnormal increases in brain connectivity. This reduction in brain hypersynchrony was associated with better cognitive performance. |
| Hötting et al. 2013 (96) | RCT | N=33 healthy adults  Mean age=48.90 years | Cognitive training (spatial vs. perceptual training) and  physical training (endurance training vs. non-endurance training) for 24 weeks | Brain activity on task-based fMRI | Only those in the spatial cognitive training group showed improved performance in the maze task. These behavioral gains were accompanied by a decrease in frontal and temporal lobe brain activity. |
| Ten Brinke et al. 2021 (97) | RCT | N=124 healthy older adults Age=65-85 years | Computerized cognitive training for 8 weeks | Functional connectivity on resting-state fMRI | Computerized cognitive training was associated with improvements in executive functions in older adults by altering the connectivity between different brain networks |
| Kang et al. 2021 (98) | RCT | N=41 older adults with subjective cognitive decline and mild cognitive impairment | Semi-immersive virtual reality-assisted cognitive training for 4 weeks. | Functional connectivity on resting-state fMRI | Virtual reality cognitive training significantly improved visuospatial abilities, reduced apathy, and enhanced overall well-and increased connectivity between the frontal and occipital regions of the brain linked to better cognitive performance. |
| Hardcastle et al. 2022a (99) | RCT | N=58 healthy older adults  Mean age= 70.67 years | Cognitive training (targeted attention/  speed-of-processing and four tasks targeted working memory from the Posit Science Brain HQ suite) for 12 weeks | Functional connectivity on resting-state fMRI | Cognitive training helped older adults perform better on various tasks, especially those requiring divided attention and speed. After the training, the frontoparietal control network showed stronger connections, and this increased connectivity was linked to better task performance. |
| Hardcastle et al. 2022b (100) | RCT | N=267 healthy older adults  Mean age= 71.68 years | Speed-of-processing abilities training | Functional connectivity on resting-state fMRI | Better connectivity (especially in the cingulo-opercular network and the frontoparietal control network), is linked to better performance on tasks that measures speed of processing and divided attention. |
| Chen et al. 2022 (101) | RCT | 49 older adults with amnesic mild cognitive impairment and 28 active controls. | Visual speed of processing cognitive training for 6 weeks | Functional connectivity on resting-state fMRI | 6-week visual speed of processing training significantly improved working memory in individuals with amnesic mild cognitive impairment compared to active controls. This improvement was linked to enhanced brain network integration. |
| Cao et al. 2016 (102) | RCT | 48 healthy older adults  Mean age= 69years  multidomain cognitive training vs. single domain cognitive training | 12 weeks of multi-domain intervention comprising memory exercises, reasoning, problem-solving/strategy, visuospatial ability, handicrafts, and physical exercise tips vs. single domain intervention with reasoning training | White mater microstructure on diffusion tensor imaging | Multi-domain cognitive training led to positive changes in white matter microstructure accompanied by improvements in processing speed. |
| Jiang et al. 2021 (103) | RCT | 32 healthy older adults  mean age=69 years | 12 weeks of multidomain cognitive training for | Structural connectivity on diffusion tensor imaging | Cognitive training showed significant improvement in delayed memory and a trend toward better overall cognitive function compared to a control group |
| Sugimoto et al. 2022 (104) | RCT | 61 healthy older adults Mean age=72 years | 12 weeks of photo‑Integrated Conversation Moderated by Robots (PICMOR) intervention vs. free conversation program | Voxel-based morphometry on structural MRI | PICMOR group showed increased brain volume in superior frontal gyrus and hippocampus in the intervention group compared to the control group. |
| Engvig et al. 2010 (106) | RCT | 42 older adults  Mean age =61.30 years | 8 weeks of cognitive training to improve verbal source memory | Cortical thickness on MRI | Memory training improved source memory and increased cortical thickness in right fusiform and lateral orbitofrontal cortex linked to better memory performance. |
| van Balkom et al. 2022 (107) | RCT | Older adults with Parkinson’s disease  Mean age=63.20 years | 8 weeks of online multi-domain cognitive training and an active control condition | Functional connectivity on resting-state fMRI | No significant overall changes in brain network connectivity or topology in individuals |
| Biel et al. 2020 (108) | RCT | 83 healthy older adults Mean age=63.93 years | 4 weeks computerized cognitive training for working memory either in combination with novel or with familiarized nature movies | Gray matter volume, myelination, and iron levels on MRI | The working memory training program improved participants' performance on the trained task but did not enhance other cognitive abilities or lead to structural changes in the brain. |
| **Cognitive training effects on BDNF levels** | | | | | |
| Jeong et al. 2016 (109) | RCT | 293 older adults with amnestic mild cognitive impairment | 12 weeks of group-based cognitive Intervention (GCI): memory training, cognitive rehabilitation, and activities to improve daily living skills using various memory strategies vs. home-based cognitive intervention | BDNF | GCI or HCI had better cognition than the controls. The changes in BDNF levels significantly correlated with the changes in the modified cognition in both GCI and HCI groups |

| **Supplementary table 4.** Randomized controlled trials on the effect of dietary intervention on dementia related biomarkers |
| --- |

| **Study** | **Design** | **Population** | **Intervention** | **Biomarker outcome** | **Findings** |
| --- | --- | --- | --- | --- | --- |
| **RCTs with neuroimaging as outcome** | | | | | |
| Barnes et al. 2023 (113) | RCT | N=267 individuals with family history of dementia, overweight, suboptimal diet Mean age= 70 years | 3 years of dietary counselling based on MIND diet with weight loss vs. weight loss only. MIND group targeted weight loss by changing content of diet, weight loss only group by changing amount of food. Complementary olive oil+nuts+blueberries provided to the MIND group | MRI measures of total, hippocampal, and white matter hyperintense lesions volumes | White-matter hyperintense volumes increased in both the MIND-diet group and the control-diet group, whereas hippocampal and total brain volumes decreased in both groups, no difference between the groups. Both groups lost weight. |
| Soininen et al. 2021 (114) | RCT | N= 311 individuals with prodromal AD  Age=55-85 years | Souvenaid multinutrient product vs. placebo for 2 years, 1-year post-intervention follow-up for 36 months | Hippocampal, ventricular, and whole brain atrophy | All brain volume changes in favour of the active intervention. The rates of deterioration for hippocampal, whole brain, and ventricular volumes 33%, 22%, and 20% less in the active group than in the control group (more pronounced differences after 24 months). |
| Arjmand et al. 2022 (115) | RCT | N=40 middle-aged, obese women without metabolic complications  Age= 40-60 years | Dietary counselling based on the MIND dietary pattern (no complementary foods) vs. weight-loss counselling for 3 months | MRI cortical thickness, surface area, and cortical volume measures | MIND diet prevented surface area loss in inferior frontal gyrys compared with weight loss. Both groups showed decrease in cerebellum white matter and cerebellum gray matter, MIND group slightly more, but without statistically significant difference between the groups. |
| Kaplan et al. 2022 (116) | RCT | N=284 individuals with abdominal obesity or dyslipidaemia  Mean age= 51 years | Healthy dietary guidelines (HDG) vs. Mediterranean diet (MED) vs. MED with supplementary foods (Green-MED); diet combined with PA in all three groups. HDG included traditional counselling only; MED aimed at calorie-restricted, traditional MED low in simple carbohydrates, including 28 g walnuts/d; Green-MED additionally aimed at avoiding processed and red meat, consuming more plant-based, drinking 3–4 cups/d of green tea, and consumption of 100 g of Wolffia globosa (Mankai) frozen plant cubes for 18 months | MRI | Lateral ventricle volume (LVV) expansion was attenuated in the Green-MED group compared with the HDG group. Significant age interactions observed: both MED diets had a significantly lower decline in hippocampal occupancy score and a smaller increase in lateral ventricle volume than HDG participants among those > 50 yrs. |
| Boraxbekk et al. 2015 (117) | RCT | N=20 women, post-menopausal, overweight or obese without self-reported cognitive impairment  Mean age=61 years | Modified paleolithic diet (PD) vs. a standard diet adhering to the Nordic Nutrition Recommendations (NNR). Counselling delivered by a dietician. Both diets were ad libitum without any calorie restrictions for 6 months | fMRI (brain responses related to episodic memory) | Significant changes in brain activation observed throughout the brain without differences between the PD and the NNR. Both the PD and the NNR also improved in anthropometric measurements and memory, without a group difference. |
| **RCTs with CSF markers as outcome** | | | | | |
| Bayer-Carter et al. 2011 (119) | RCT | N=49 individuals with normal cognition, and amnestic MCI  Mean age= 68 years | 4 weeks of diet high in fat and glycemic index (HIGH) vs. diet low in fat and glycemic index (LOW). The HIGH aimed at 45 E% fat, >25 E% saturated fat, 35–40 E% carbohydrates, 15%–20% protein; the LOW diet aimed at 25 E% fat, <7 E% saturated fat, 55–60 E% carbohydrates, 15%–20% protein. | CSF: Aβ42 and Aβ40, tau, insulin, F2-isoprostanes, and apolipoprotein E | For aMCI group, the LOW diet increased CSF Aβ42, whereas LOW diet had the opposite effect for healthy adults by decreasing Aβ42, and HIGH diet increasing Aβ42. The CSF apolipoprotein E concentration was increased by the LOW and decreased by the HIGH diet for both groups. |
| Hanson et al. 2013 (120) | RCT | N=47 individuals with normal cognition, and amnestic MCI  Mean age= 68 years | 4 weeks of diet high in fat and glycemic index (HIGH) vs. diet low in fat and glycemic index (LOW). The HIGH aimed at 45 E% fat, >25 E% saturated fat, 35–40 E% carbohydrates, 15%–20% protein; the LOW diet aimed at 25 E% fat, <7 E% saturated fat, 55–60 E% carbohydrates, 15%–20% protein. | CSF lipid-depleted (LD) Aβ40, Aβ42, and apolipoproteins. | No significant differences but The LOW diet tended to decrease LD Aβ levels, whereas the HIGH diet increased. |
| **RCT with blood markers as outcome** | | | | | |
| Sánchez-Villegas et al. 2011 (126) | RCT | N=243  men aged 55-80 and women aged 60-80 years with increased CVD risk | 3 years of dietary interventions: 1) low fat (control) 2) Mediterranean Diet (MeDi) + supplementary virgin olive oil 3) MeDi + supplementary nuts. No energy restrictions. | BDNF | Plasma BDNF levels were not higher for participants assigned to MeDi combined,but those in MeDi + Nuts showed a significant lower risk of low plasma BDNF than the control group. |
| **RCT with multiple outcomes (MRI and CSF)** | | | | | |
| Hoscheidt et al. 2021 (118) | RCT | N=87  age range 45-65  normal cognition (CN, n=56) & MCI (n=31) | 4 weeks of mediterranean type (Med) vs. Western type (West) diet. Med aimed at low saturated fat (SF), low glycemic index (GI), and low sodium (Na+) (40% total fat; <7% SFA; 40-45 % carbs; 15-20 % protein), and West at high SF, high GI, high Na+ (40-45% total fat; 25% SFA; 40 % carbs;15-20 % protein). | CSF: amyloid beta (Aβ)42, total and phosphorylated tau (t-tau, p-tau181); MRI: MP-RAGE, pcASL | CFS: For the NC participants, CSF Aβ40 decreased with Med-diet and increased with West-diet. No effects for CSF Aβ42, but a three-way interaction of diet x cognitive group x time was observed for the CSF Aβ42/40 ratio, which increased in Med-diet and decreased in West-diet in NC. For the MCI group t-tau increased by the Med-diet and decreased by the West-diet. t-tau was unchanged by either diet in NC group. MRI: Cerebral perfusion (CB) increased with Med-diet and decreased with West-diet, only in NC participants. No effects in MCI group. |
| **Non-randomised trial with blood marker as outcome** | | | | | |
| Nilholm et al. 2018 (275) | Non-randomised trial | N=30  mean age 58  type 2 diabetes | 12 weeks (28 wk follow-up) of okinawan-based Nordic diet (O-BN) with two meals a day, and snacks consisting of a variety of fruits, berries, and seeds. The O-BN diet was based on the traditional Okinawan diet but modified to suit the Nordic population. | NfL, inflammatory markers | Intervention resulted in increase in NfL and improved inflammatory parameters (lowered IL-18) at 12 wk, but not in longer follow-up after 28 weeks. |

| **Supplementary table 5.** Randomized controlled trials on the effect of diabetes type 2 interventions on dementia related biomarkers | | | | | |
| --- | --- | --- | --- | --- | --- |
| **Study** | **Design** | **Population** | **Intervention/Predictor** | **Biomarker outcome** | **Findings** |
| **Meta analyses of observational studies with neuroimaging outcomes** | | | | | |
| Moulton et al. 2015 (131) | MA of Observational studies | N = Ten T1DM studies (n = 613 patients) and 23 T2DM studies (n = 1364 patients)  Age = 25.8 years (T1DM) 63.2 years (T2DM) | DM | Brain volume/atrophy | The T1DM meta-analysis revealed reduced bilateral thalamus grey matter density in adults  The T2DM meta-analysis revealed reduced global brain volume and regional atrophy |
| Wu et al. 2017 (132) | MA of Observational studies | N = 15 volumetric studies and five VBM (Voxel-Based Morphometry); n = 1561 T2DM, n = 869 HC volumetric studies; n = 110 T2DM, n = 103 HC VBM  Age = 53+ y | T2DM | Grey matter changes | The volumetric meta-analysis showed that the GMV (Global gray matter volumes) of patients with T2DM is lower than in HCs. The whole-brain meta-analysis revealed GM reductions in T2DM patients compared with HCs. Meta-regression analysis showed that Mini-Mental State Examination (MMSE) scores have a positive relationship with GMV |
| Yao et al. 2021 (133) | MA of Observational studies | N = 15 structural datasets (693 patients and 684 controls) and 16 functional datasets (378 patients and 358 controls)  Age = 55+ | T2DM | Grey matter volume | In patients with T2DM compared to controls, decreased regional gray matter volume and altered intrinsic activity |
| Zhou et al. 2021 (134) | Coordinate‐Based Meta‐Analysis | N = 8 studies  Age = 16+ y | T2DM | DTI | FA reductions in the left inferior network, the corpus callosum, and the left olfactory cortex. FA in the CC was negatively correlated with BMI in the patients group. |
| **RCTs with neuroimaging as outcome** | | | | | |
| Erus et al. 2015 (136) | RCT | N=488  Mean age =62.2 ± 5.6 y | Intensive glycemic treatment targeting HbA1c to less than 6.0% or standard glycemic treatment targeting HbA1c to 7.0–7.9%.  Duration: 3.5 years | Grey and white matter volume | Decelerated loss of gray matter volume associated with intensive glycemic treatment. No significant relationship between low versus high baseline HbA1c levels and brain changes was found. Regions in which cognitive change was associated with longitudinal volume loss had only small overlap with regions related to diabetes duration and to treatment effects. |
| Zhang et al. 2015 (135) | RCT | N = 14 diabetic and 14 healthy subjects  Age = Diabetic 61.7 years and Healthy subjects 60.19 years | Single dose of intranasal insulin or sterile saline (placebo) in T2DM and healthy older adults | Enhanced Resting-State Functional Connectivity of Hippocampal Regions | Following insulin administration, diabetic patients demonstrated increased resting-state connectivity between the hippocampal regions and the medial frontal cortex (MFC) as compared with placebo and other DMN regions. |
| Espeland et al. 2016 (139) | RCT | N = 319  Age = 45-76 | Diet modification and physical exercise  Duration: 9.8 ± 0.7y for ILI participants (MRI 0.6 ± 0.7y) and 9.9 ± 0.7y (MRI 0.5 ± 0.8y) for DSE participants | Brain and White Matter Hyperintensity Volumes | Total brain and hippocampus volumes were similar between intervention groups. The mean white matter hyperintensity volume was 28% lower among lifestyle intervention participants compared with those receiving diabetes support and education. |
| **RCTs with cerebral blood flow and oxygenation as outcome** | | | | | |
| Sato et al. 2011 (137) | Open label RCT | N = 42  Mean age= 77.6 years | Treatment: 15–30 mg pioglitazone daily (n = 21, pioglitazone group) or not (n = 21, control group)  Duration: 6 months | rCBF | The pioglitazone group improved cognition and rCBF in the parietal lobe, while the control group showed no such improvement. Both groups showed good control of diabetes during the study. |
| **RCTs with Blood/CSF Biomarkers as outcome** | | | | | |
| Fishel et al. 2005 (140) | RCT | N=16  Mean age = 68.2 years | On separate mornings, fasting participants received randomized infusions of saline or insulin with variable dextrose levels to maintain euglycemia, achieving plasma insulin levels typical of insulin resistance.  Duration: By at least 1 week (mean interval 19.3 days, SD 3.5 days) | Interleukin 1α, interleukin 1β, interleukin 6, tumor necrosis factor α, F2-isoprostane (CSF only), Aβ, norepinephrine, transthyretin, and apolipoprotein E | Insulin increased CSF levels of F2-isoprostane and cytokines as well as plasma and CSF levels of Aβ42. The changes in CSF levels of Aβ42 were predicted by increased F2-isoprostane and cytokine levels and reduced transthyretin levels. Increased inflammation was modulated by insulin-induced changes in CSF levels of norepinephrine and apolipoprotein E. |
| Watson et al. 2005 (144) | RCT | N=30  Age range = 55-85 years | Rosiglitazone (4 mg daily; N = 20) or placebo (N = 10)  Duration: 6 months | Plasma Aβ | Subjects receiving rosiglitazone exhibited better delayed recall (at Months 4 and 6) and selective attention (Month 6). At Month 6, plasma Aβ levels were unchanged from baseline for subjects receiving rosiglitazone but declined for subjects receiving placebo, consistent with recent reports that plasma Aβ42 decreases with progression of AD. |
| Sato et al. 2011 (137) | Open label RCT | N = 42  Mean age= 77.6 years | Treatment: 15–30 mg pioglitazone daily (n = 21, pioglitazone group) or not (n = 21, control group)  Duration: 6 months | Plasma levels of Aβ40 and Aβ42 | The pioglitazone group improved cognition. The plasma Aβ40/Aβ42 ratio increased in the control group but showed no significant change in the pioglitazone group. |

| **Supplementary table 6.** Randomized controlled trial on the effect of obesity intervention on dementia related biomarkers |
| --- |

| **RCT effects on fluid biomarkers** | | | | | |
| --- | --- | --- | --- | --- | --- |
| Horie et al. 2016 (161) | Prospective controlled trial | N= 80 obese patients with MCI  *75 completed the Follow-up  Age: ≥60 | Random allocation (1:1)  conventional medical care alone n = 40  Nutritional counseling n= 40  Group meetings (by nutritionist)  Duration= 12 months | Serum levels of Leptine, HOMA-IR,  CRP | Changes in metabolic markers and diet following intervention were associated with improvement in cognitive tests. |

| **Supplementary Table 7.** Randomized controlled trials on the effect of hypertension interventions on dementia related biomarkers | | | | | |
| --- | --- | --- | --- | --- | --- |
| **Study** | **Design** | **Population** | **Intervention/Predictor** | **Biomarker outcome** | **Findings** |
| **Systematic reviews and Meta-analysis of Observational studies with neuroimaging as outcomes** | | | | | |
| Brown et al. 2021 (169) | SR and MA of observational studies | N studies = 59 | Hypertension | White matter hypersensitivity | WMH growth rate was lower in patients with non-stroke cardiovascular disease, and with cognitive impairment. |
| Beauchet et al. 2013 (171) | SR and MA of observational studies | N = 28 studies  Age = mean age ranged from 45.4 ± 15.7 to 81.5 ± 5.0 years | Hypertension | Brain atrophy | No between-group difference regarding the whole-gray matter volume. Cases with hypertension exhibited lower hippocampus volume compared with controls. |
| Ma et al. 2020 (170) | SR and MA of observational studies | N = 2796 (10 independent population-based prospective cohort studies)  Age = 74 ± 4 y | Blood pressure | White matter hypersensitivity | Increase in systolic BP variability was associated with increased odds of the presence or progression of white matter hyperintensities. The association of systolic BP variability with the presence of lacunes and the presence of microbleeds were not statistically significant |
| Gutteridge et al. 2022 (172) | SR | N studies = 20 | Blood pressure | Brain volume/atrophy | Associations between MRI indices and BP dipping patterns were mixed; higher long-term BPV and higher sleep systolic BPV was found to be associated with lower whole brain volume and hippocampal volume. |
| **Systematic reviews and Meta-analysis of RCTs with neuroimaging as outcomes** | | | | | |
| Lai et al. 2020 (182) | 7 RCTs | N = Seven studies with 2693 patients  Age = Median age of 67.1 years (range: 60.8-80.6 years | Anti-hypertensive treatment | White matter hypersensitivity | Intensive BP control prevents WMH progression, and its effect is associated with the magnitude of intensive BP control. |
| **RCTs with neuroimaging as outcome (not included in the meta-analyses above)** | | | | | |
| Firbank et al. 2007 (176) | RCT | N = 163  Age = 70–89 years | Treatment: Candesartan cilexetil (intervention) or control (placebo) | Brain atrophy and white matter hyperintensity | Total WMH fraction increased in both normotensive and treated hypertensive groups median change. Deep WMH increased in hypertensive but not the normotensive group. Regression analysis found significant predictors of change in WMH to be blood pressure and initial deep WMH, but not treatment group. |
| **RCTs with cerebral blood flow and oxygenation as outcome** | | | | | |
| Jennings et al. 2010 (174) | Uncontrolled Trial (ppts tested pre and post treatment) | N = 43  Age = 52.5 years | Treatment: Lisinopril/atenolol  (absence of an untreated or placebo control group)  Duration: 1 year | rCBF | Neuropsychological performance improved over the year of treatment but was unrelated to change in regional cerebral blood flow (rCBF). Neither mean resting rCBF nor responsivity to a working memory task changed significantly with treatment. |
| Jennings et al. 2008 (173) | Uncontrolled Trial (comparison of two antihypertensive treatments) | N = 28  Age = 52 years | 1 year treatment of Lisinopril/atenolol  (absence of an untreated or placebo control group) | rCBF brachial artery diameter | Pharmacologic treatment of hypertension (with either lisinopril or atenolol) did not normalize regional cerebral blood flow (rCBF) responses to memory processing or acetazolamide injection. There were no significant differences in the magnitude of rCBF responses pre- and post-treatment. |
| Jiang et al. 2023 (180) | RCT | N = 8563 participants (4278 in the intensive group and 4285 in the standard group)  Age = 67.9 years | 3-year treatment with intensive or standard SBP control | CBF | The effect of intensive treatment on cerebral blood flow was not modified by baseline DBP. Even among participants within the lowest DBP quartile, intensive versus standard BP treatment resulted in an increasing trend of annualized change in cerebral blood flow |
| Kume et al. 2012 (175) | RCT (randomized, open-label parallel) | N = 20  Age = 79.0 years | 6 months of treatment with telmisartan or amlodipine  Duration | rCBF | The groups had a similar significant reduction in systolic and diastolic blood pressure after treatment.  Analysis of covariance to analyze treatment effect revealed that the telmisartan group showed increased rCBF in the right supramarginal gyrus, superior parietal lobule, cuneus, and lingual gyrus compared with the amlodipine group, while the amlodipine group showed increased rCBF only in the right cingulate gyrus compared with the telmisartan group at 6 months. |
| **RCTs with Blood/CSF based biomarkers** | | | | | |
| Wharton et al. 2013 (183) | RCT | N = 14  Age = 54 years | 4-month treatment with Ramipril 5mg | CSF (e.g., Aβ1–42) | While results did not show a treatment effect on CSF Aβ1–42 (p=0.836), data revealed that ramipril can inhibit CSF ACE activity (p=0.009) and improve blood pressure (BP), however there were no differences between groups in arterial function or cognition.  In this study, ramipril therapy inhibited CSF ACE activity and improved BP, but did not influence CSF Aβ1–42. |

| **Supplementary Table 8.** Randomized controlled trials on the effect of hypercholesterolemia interventions on dementia related biomarkers | | | | | |
| --- | --- | --- | --- | --- | --- |
| **Study** | **Design** | **Population** | **Intervention** | **Biomarker outcome** | **Findings** |
| **RCTs with neuroimaging as outcome** | | | | | |
| Smit et al. 2016 (190) | RCT | N = 535 adults with risk for developing vascular disease  Mean age =75 years | 3.2 years of follow-up after treatment with Pravastatin (40 mg/day) | Hippocampal volume, cerebral blood flow (CBF), WMHL | Higher LDL variability was associated with lower CBF in both placebo and treatment groups. In pravastatin group high LDL variability was associated with higher WMHL. No association between LDL-C variability and hippocampal volume. |
| Tendolkar et al. 2012 (191) | RCT | N = 34 elderly atrial fibrillation patients without history of stroke or severe neurological conditions and diabetes.  Mean age = 74 years | 1 year of treatment with Atorvastatin (20 mg/day) 2 weeks 🡪 Atorvastatin (40 mg/day) 4 weeks 🡪 Atorvastatin (40 mg/day) + ezetimibe (10 mg/day) [on top of anticoagulant treatment] | MRI  Amygdala and hippocampal volume, WML | Placebo group exhibited more atrophy for right amygdala and left hippocampus. |
| Carlsson et al. 2012 (192) | RCT | N = 16 cognitively asymptomatic adults with parental history of AD  Age = 38–66 years | 4 months of treatment with Atorvastatin (40 mg/day) | MRI  Cerebral blood flow | Atorvastatin group showed a greater increase in regional CBF in bilateral hippocampi, fusiform gyrus, putamen and insular cortices compared to participants on placebo. Global CBF changes were not significantly different between the groups |
| Zhang et al. 2019 (193) | RCT | N = 732 hypertensive, non-demented elderly patients  Age = mean 70.7 years | 59.8 months of follow-up after treatment with Rosuvastatin (10 mg/day) + open label hydrochlorothiazide | MRI  WMH | Rosuvastatin use was associated with lower risks of new-incident Fazekas scale scores ≥2 and lower risk of WMH progression |
| Vogt et al. 2021 (194) | RCT | N = 73 cognitively asymptomatic adults with parental history of AD  Mean age=56 years | 18 months of treatment with simvastatin (40 mg/day) | MRI  WM microstructure, volume and WMH | Total WM volume was preserved in the treatment group. Treatment group showed a significant preservation in global WM as measured by fractional anisotropy and radial diffusivity. |
| Mok et al. 2009 (195) | RCT | N = 208 cognitively normal adults  Age = median 63 years | 2 years of treatment with **s**imvastatin (20 mg/day) | MRI  white matter lesion (WML) | Statin treatment reduced WML progression in subjects with severe WML at baseline. Statin treatment independently predicted change in WML volume. No significant change in WML volume between the active and the placebo group at 2 years |
| Taylor et al. 2018 (196) | RCT | N = 150 healthy adults  Age = mean 48 years | 6 months treatment with Atorvastatin (80 mg/day) | fMRI  BOLD, activation of different brain regions | Participants on atorvastatin had greater activation in the bilateral precuneus but the effect was reversed after drug washout |
| **RCTs with ADRD fluid biomarkers as an outcome** | | | | | |
| Li et al. 2017 (197) | RCT | N = 49 statin-naive with normal cognition and normal or mildly elevated cholesterol  Age = 45-64 years | 1 year treatment with simvastatin (40 mg/day) | CSF Ab42, total tau, and p-tau181 | No differences in change in CSF markers between treatment groups. |
| Carlsson et al. 2008 (198) | RCT | N = 57 cognitively asymptomatic adults with parental history of AD and high prevalence of ApoE4  Age = 40–65 years | 4 months of treatment with simvastatin (40 mg/day) | CSF  Ab42, total tau | No difference between simvastatin and placebo. |
| Riekse et al. 2006 (199) | Randomized, blinded, treatment trial (no placebo) | N = 24 individuals with normal cognition and hypercholesterolemia  Age = 34–87 years | 3 months of treatment with simvastatin (40 mg/day) or pravastatin (80 mg/day) | CSF Aβ40, Aβ42, t-tau, p-tau181, sAβPPα, sAβPPβ | p-tau levels decreased with simvastatin but not pravastatin treatment. No changes in Aβ, t-tau, or sAPPs |
| Friedhoff et al. 2001 (200) | RCT | N = 172 individuals meeting the criteria for HMG-CoA reductase inhibitor treatment and without comorbid medical conditions  Age = 29–70 years | 12 weeks of treatment with lovastatin (10, 20, 40 or 60 mg/day) | Serum Aβ | Statin treatment decreased levels of Aβ in a dose-dependent manner |
| Ishii et al. 2003 (201) | Treatment trial (no placebo) | N = 46 non-demented individuals with hyperlipidaemia, hypertension and/or diabetes  Age = 33-83 years | 3–6 months of treatment with pravastatin (10 mg/d) | Plasma Aβ42, Aβ40 | Plasma Aβ levels were unchanged. No association between change in cholesterol levels and Aβ species |
| Tokuda et al. 2001 (202) | Placebo controlled treatment trial | N = 45 cognitively normal subjects  Age = approx. 62 years | Treatment with Pravastatin (10 mg/day) or Simvastatin (5 mg/day) | Plasma Aβ42, Aβ40 | No differences observed in Aβ levels between groups. |
| Höglund et al. 2004 (203) | Prospective, randomized, dose-finding treatment trial (no placebo) | N = 39 hypercholesterolemic individuals without cognitive impairment  Mean age=55 years | 36 weeks of treatment with simvastatin (40 mg/d), or atorvastatin (20 mg/d) for 6 weeks; followed by simvastatin (80 mg/d) or atorvastatin (40 mg/d) for 6 weeks; finally, simvastatin (80 mg/d) or atorvastatin (80 mg/d) for 24 weeks | Plasma Aβ40, Aβ42, total Aβ | No changes in Aβ levels during statin treatments |
| Sparks et al. 2005 (204) | RCT | N = 63 including MCI and AD patients (longitudinal part)  Mean age = 78.5 years | 1 year treatment with atorvastatin (80 mg/day) | Plasma Aβ40 and Aβ42 | Non-significant increase in Aβ markers in statin group. |
| Wei et al. 2022 (205) | RCT | N = 120  Mean age = 57 years | 12 weeks treatment with simvastatin (40 mg/day) | Plasma Aβ40, Aβ42, Aβ42/Aβ40 ratio, sLRP1, and sRAGE | Small differences observed between the placebo and treatment group |
| **RCTs with MRI and inflammatory markers as outcome** | | | | | |
| Lappegård et al. 2013 (206) | RCT | N = 34  Mean age= 74 years | See above | MRI Brain volume, inflammatory markers in plasma | Volume loss in left amygdala was slower in treatment group compared to placebo. Reduction in inflammatory markers. |

| **Supplementary Table 9.** Randomized controlled trials on the effect of sleep or stress-reducing interventions on dementia related biomarkers | | | | | |
| --- | --- | --- | --- | --- | --- |
| **Study** | **Design** | **Population** | **Intervention** | **Biomarker outcome** | **Findings** |
| **RCTs with neuroimaging as outcome** | | | | | |
| Ueno-Pardi et al. 2022 (219) | RCT | N=47 adults with obstructive sleep apnea  Age: controls 51 years, intervention 53 years, 57% male | 6 months of aerobic cycling and strength components containing exercises, 3 sessions/week, 60 min/session | Regional cerebral metabolic glucose rate (CMRgl) | Exercise intervention increased CMRgl in the right frontal lobe (p<0.05). CMRgl was inversely associated with OSA severity and attention/executive functioning after intervention. |
| McCrae et al. 2018 (220) | RCT | N=37 participants with fibromyalgia and insomnia  Mean age=55.9 years | 8 weeks cognitive behavioral therapy, 3 groups: sleep education, pain education and controls | Cortical thickness | Intervention effect on left lateral orbitofrontal and left rostral middle frontal cortex. Pairwise comparison showed cortical thinning in control and pain education group and thickening in insomnia education group. Increase in cortical thickness was associated with reduction in wake after sleep onset in sleep education group. |
| Altena et al. 2008 (221) | RCT | N=33, 73 % female  Mean age=60 years | 6 weeks sleep therapy: chronic insomnia patients (therapy and control group) and healthy matched controls | fMRI during letter and category fluency tasks and counting backwards | Insomnia patients showed hypoactivation of the medial and inferior prefrontal cortical areas which recovered after successful sleep therapy. |
| Killgore et al. 2020 (223) | RCT | N=32 participants with mild traumatic brain injury, 56.2% female  Mean age= 23 years | 6-weeks of daily 30-min pulses of blue light each morning versus amber placebo light | Neuroimaging (structural MRI), connectivity, diffusion tensor imaging (DTI) | The blue light intervention in patients with mild traumatic brain injury was associated with increases in gray matter volume within the posterior thalamus and greater structural and functional thalamocortical connectivity. |
| **RCT with BDNF as outcome** | | | | | |
| Alikhani et al. 2020 (225) | RCT | N=75 HIV male patients receiving methadone Mean age =39.6 years | 12 weeks, 3 groups: trazodone, weekly sleep hygiene training or weekly sleep hygiene training and trazodone | BDNF | BDNF did not change significantly or descriptively over time, between the groups, or between groups over time. |

| **Supplementary Table 10.** Randomized controlled trials on the effect of multimodal interventions on dementia related biomarkers | | | | | |
| --- | --- | --- | --- | --- | --- |
| **Study** | **Design** | **Population** | **Intervention** | **Biomarker type** | **Main findings** |
| **RCTs with neuroimaging as outcome** | | | | | |
| Stephen et al. 2019 (237) | RCT | N=1260, community-dwelling older adults 60-77 years, at risk of dementia (CAIDE risk score and cognitive performance).  MRI Sub-study N=112 | 2-year multimodal lifestyle intervention (diet, exercise, cognitive training, vascular risk monitoring) vs. general health advice | Neuroimaging (structural MRI) | No statistically significant group differences in 2-year changes in MRI measures. Beneficial intervention effects on processing speed more pronounced in those with higher baseline cortical thickness in AD signature areas and a similar trend for higher hippocampal volume |
| Stephen et al. 2020 (239) | See above | See above.  MRI Sub-study N=60 | See above | Neuroimaging (structural MRI) | FA decreased, and cognition improved more in the intervention group than in the control group. No statistically significant group differences for changes in MD, AxD, or RD. |
| Sivera et al. 2020 (240) | RCT | N=1680; community-dwelling older adults 70+ years, at risk of dementia (memory complaint, iADL limitations, or slow gait speed).  MRI Sub-study N=376 (baseline and 3-year MRI) | 3-year intervention, 4 groups: multimodal lifestyle intervention (MI) + omega 3 supplement; MI + placebo; omega-3 alone; placebo alone | Neuroimaging (structural MRI) | Statistically significant effects of the MI but not omega-3 on the MRI measures, observed mainly in the left periventricular area near the [temporoparietal junction](https://www.sciencedirect.com/topics/medicine-and-dentistry/temporoparietal-junction). Changes associated with better cognitive performance. |
| Delrieu et al. 2020 (241) | See above | See above.  FDG-PET sub-study N=67 (baseline; N=58 at follow-up). 1:1 inclusion of participants in the MI groups and no MI groups. | See above. | Neuroimaging (FDG-PET) | No statistically significant effect of the MI on brain glucose metabolism. Certain shorter-term (6 months) but not longer-term (12 months) favourable intervention effects on the change of cerebral glucose metabolism |
| Perus et al. 2023 (242) | See above | See above.  MRI Sub-study N=100 | See above. | Neuroimaging (rs-fMRI) | Overall no group differences in the FC changes. Certain FC differences observed between groups with and without omega-3 supplementation if baseline cognitive status (CDR) was considered. |
| Andrieu et al. 2017 (20)  Delrieu et al. 2019  (243) | See above | See above.  Amyloid PET Sub-study N=269 | See above. | Neuroimaging (amyloid PET) | Beneficial intervention effects in the amyloid positive group but not in negative. |
| van Dalen et al. 2017 (244) | RCT | N=3526; individuals aged 70-78 years through participating general practices (cluster randomization).  MRI Sub-study N=195 without dementia and systolic BP ≥140 mmHg  Longitudinal analysis: N=126. | 6-year nurse-led, multimodal cardiovascular intervention or control (usual care) | Neuroimaging (structural MRI) | Annual WMH volume increase was similar in the intervention and control groups. No group difference in the development of new lacunar infarcts (overall, only few cases occurred).  Greater intervention effects with increasing baseline WMH volumes. |
| Lenze et al. 2022  (245) | RCT | N=585 older adults (65-84 years) with subjective cognitive concerns but no dementia | 18-month program; 1:1:1:1 Mindfulness-based stress reduction (MBSR) with a daily target of 60 min meditation; exercise with aerobic, strength, and functional components with a weekly target of >= 300 min; combined MBSR and exercise; or a health education control group. | Neuroimaging (structural MRI) | For the combined intervention group, no intervention effects on MRI measures were observed at 6 or 18 months. |
| Castells-Sánchez et al. 2022 (246) | RCT | N=109 (analyses conducted per protocol N=82), healthy adults aged 50-70 years, no substantial cognitive impairment. | 3-month intervention, 4 groups:  Aerobic exercise (progressive brisk walking program), computerized cognitive training CCT, AE + CCT, and control (asked not to alter regular lifestyle). | Neuroimaging (structural MRI), blood biomarkers | No statistically significant changes between the groups in any outcomes. |
| Train the Brain Consortium 2017. (247) | RCT | N=113 aged between 65-89 years with MCI  N=70 with repeated structural MRI, fMRI N=50 | 7-month intervention (cognitive training, physical exercise and music therapy) vs. control (asked to continue with their usual life routine). | Neuroimaging (structural MRI, fMRI) | No statistically significant group differences in hippocampal volumes. Significant increase in CBF in favor of the intervention group in the parahippocampal regions.  fMRI: increase of BOLD signal in the control group, suggesting potentially a reduced neural efficiency (preserved in the intervention group). |
| Moon et al., 2022  (248) | RCT | N=152, individuals aged 60–79 years, at-risk of dementia.  Sub-study N=55 | 24-week intervention, 3 groups, 1:1:1 facility-based (FMI) or home-based (HMI) FINGER-style intervention vs. control regular health advice | Neuroimaging (structural MRI), blood markers | Positive intervention effects (for FMI) on mean global cortical thickness and particularly thickness of the bilateral frontotemporal lobes, cingulate gyri, and insula). Higher BDNF levels in FMI vs. control. |
| Moon et al. 2022. (249) | See above | See above.  Sub-study N=56 | See above. | Neuroimaging (fMRI), blood markers | No significant changes in BDNF levels among groups (statistically non-significant interaction term)  Changes in ReHo: Observed in both intervention groups (different areas) but not in control. Some group differences, in the right cuneus (HMI vs. control), and in the left medial orbitofrontal gyrus and the right superior parietal lobule (FMI vs. control). In FMRI, these changes correlated with BDNF changes and to some extent with cognitive changes. |
| Lee et al. 2023  (250) | See above | See above.  Sub-study N=55 | See above. | Neuroimaging (structural MRI) | Within-group longitudinal change in FA, MD, AD, and RD. Mostly non-significant group differences in these longitudinal changes, except for AD in the CgC where a beneficial intervention effect was observed. AD changes of the CgC correlated with BDNF but not cognitive changes. |
| Bae et al. ~~J Clin Med.~~ 2020 (251) | RCT | N=280 older adults 70+ with global cognitive decline (MMSE 21-24). | 10-month single-blinded RCT of multicomponent exercise vs. control  Multicomponent: weekly 90-minute classes of aerobic exercise and cognitive combined dual-task training, strength training, and balance.  Control group: three health education classes (90 min each). | Neuroimaging (structural MRI) | Increase in cortical thickness in the intervention but not the control group; statistically significant interaction term found for the left middle temporal and left temporal pole region. Correlation with cognition. |
| Park et al. 2022  (252) | RCT | EEG Sub-study N=127 | SUPERBRAIN RCT (see above). | EEG | Intervention group had increases in the iCoh of the alpha1 band and in the relative power of the beta1 band and the absolute power of the beta3 band. Decrease in the characteristic path length of alpha1 band having associations with cognitive changes. |
| **RCTs with fluid biomarkers as outcome** | | | | | |
| Sandebring-Matton et al. 2021  (255) | RCT | Sub-study N=47 | FINGER RCT (see above) | oxysterols | Intervention-related 27-OH reduction was associated with improvement in cognition (especially memory). Not observed in control. At baseline, higher 27-OH was associated with lower total gray matter and hippocampal volume, and lower cognitive scores. |
| Håkansson et al. 2021, conference abstract (257) | RCT | Sub-study N=151 (only intervention group) | FINGER RCT (see above) | BDNF | Positive association between serum proBDNF levels at baseline and improved memory after the FINGER intervention. |
| Smith et al. 2020  (111) | RCT | N=132 sedentary older adults with CVD risk factors and cognitive impairment no dementia (CIND) (vascular cognitive impairment). | 6-month intervention, 2×2 factorial design. Aerobic exercise (AE), DASH diet, both AE+DASH, or a health education control condition. | metabolic biomarkers and neurotrophins | The combined intervention group showed the  the largest improvement in metabolic markers.  No group differences / intervention effects for the inflammation markers or neurotrophins. |
| Anderson-Hanley et al. 2018 (258) | RCT | N=111 community-dwelling older adults mean age 78, 75% MCI based on MoCA < 26.  Exploratory biomarker analyses with small sub-samples. | 6-month intervention, 3 groups: (1) exer-tour: exercise interactive with relatively passive, low cognitive load, virtual scenic bike tour; (2) exer-score: exercise interactive with a relatively effortful, high cognitive demand, videogame; (3) game-only: same videogame operated by a joystick or keyboard (no exercise required). | VEGF, BDNF miRNA-9 | Increasing VEGF correlated with better memory performance. Exercise dose correlated significantly with increase in BDNF. miRNA-9 expression correlated with improved executive functioning (exosome analysis). |
