# Supplementary material for: Mechanisms of interventions targeting modifiable factors for dementia risk reduction
Source: Mol Neurodegener. 2025 Jun 23;20:75. doi: 10.1186/s13024-025-00845-w (PMC12186355; doi:10.1186/s13024-025-00845-w)
Supplement: Supplementary file 2 — Supplementary Material 2: Supplementary Figure 1. Overview of the number of randomized controlled trials conducted for the different lifestyle intervention domains included in this reviewand total numbers of participants included in these [file 13024_2025_845_MOESM2_ESM.pdf]

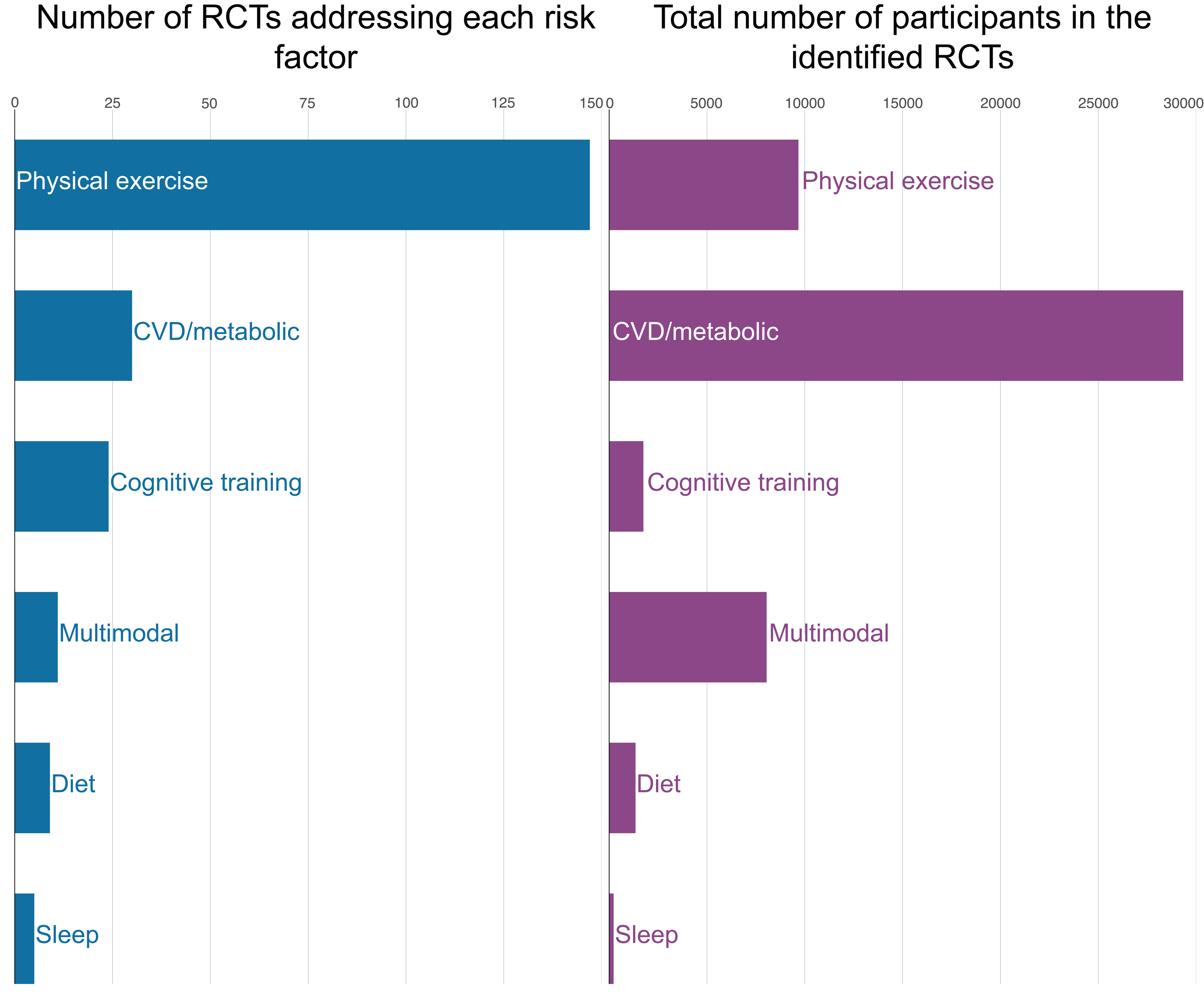

Number of RCTs addressing each risk factor
0
25
50
75
100
125
150
Physical exercise
CVD/metabolic
Cognitive training
Multimodal
Diet
Sleep
Total number of participants in the identified RCTs
0
5000
10000
15000
20000
25000
30000
Physical exercise
CVD/metabolic
Cognitive training
Multimodal
Diet
Sleep
